# Genomic Insight into Symbiosis-Induced Insect Color Change by a Facultative Bacterial Endosymbiont, “*Candidatus* Rickettsiella viridis”

**DOI:** 10.1128/mBio.00890-18

**Published:** 2018-06-12

**Authors:** Naruo Nikoh, Tsutomu Tsuchida, Taro Maeda, Katsushi Yamaguchi, Shuji Shigenobu, Ryuichi Koga, Takema Fukatsu

**Affiliations:** a Department of Liberal Arts, the Open University of Japan, Chiba, Japan; b Graduate School of Science and Engineering, University of Toyama, Toyama, Japan; c NIBB Core Research Facilities, National Institute for Basic Biology, Okazaki, Japan; d National Institute of Advanced Industrial Science and Technology, Tsukuba, Japan; e Department of Biological Sciences, Graduate School of Science, University of Tokyo, Tokyo, Japan; f Graduate School of Life and Environmental Sciences, University of Tsukuba, Tsukuba, Japan; University of Hawaii at Manoa

**Keywords:** *Acyrthosiphon pisum*, *Coxiella*, *Legionella*, *"Candidatus* Rickettsiella viridis", aphid, facultative symbiont, genome, insect body color, polycyclic quinone pigments, polyketide synthase, type IV secretion system

## Abstract

Members of the genus *Rickettsiella* are bacterial pathogens of insects and other arthropods. Recently, a novel facultative endosymbiont, “Candidatus Rickettsiella viridis,” was described in the pea aphid Acyrthosiphon pisum, whose infection causes a striking host phenotype: red and green genetic color morphs exist in aphid populations, and upon infection with the symbiont, red aphids become green due to increased production of green polycyclic quinone pigments. Here we determined the complete genome sequence of the symbiont. The 1.6-Mb circular genome, harboring some 1,400 protein-coding genes, was similar to the genome of entomopathogenic Rickettsiella grylli (1.6 Mb) but was smaller than the genomes of phylogenetically allied human pathogens Coxiella burnetii (2.0 Mb) and Legionella pneumophila (3.4 Mb). The symbiont’s metabolic pathways exhibited little complementarity to those of the coexisting primary symbiont Buchnera aphidicola, reflecting the facultative nature of the symbiont. The symbiont genome harbored neither polyketide synthase genes nor the evolutionarily allied fatty acid synthase genes that are suspected to catalyze the polycyclic quinone synthesis, indicating that the green pigments are produced not by the symbiont but by the host aphid. The symbiont genome retained many type IV secretion system genes and presumable effector protein genes, whose homologues in L. pneumophila were reported to modulate a variety of the host's cellular processes for facilitating infection and virulence. These results suggest the possibility that the symbiont is involved in the green pigment production by affecting the host’s metabolism using the secretion machineries for delivering the effector molecules into the host cells.

## IMPORTANCE

Insect body color is relevant to a variety of biological aspects such as species recognition, sexual selection, mimicry, aposematism, and crypsis. Hence, the bacterial endosymbiont “Candidatus Rickettsiella viridis,” which alters aphid body color from red to green, is of ecological interest, given that different predators preferentially exploit either red- or green-colored aphids. Here we determined the complete 1.6-Mb genome of the symbiont and uncovered that, although the red-green color transition was ascribed to upregulated production of green polycyclic quinone pigments, the symbiont genome harbored few genes involved in the polycyclic quinone biosynthesis. Meanwhile, the symbiont genome contained type IV secretion system genes and presumable effector protein genes, whose homologues modulate eukaryotic cellular processes for facilitating infection and virulence in the pathogen Legionella pneumophila. We propose the hypothesis that the symbiont may upregulate the host’s production of polycyclic quinone pigments via cooption of secretion machineries and effector molecules for pathogenicity.

## INTRODUCTION

Insects represent a substantial portion of the terrestrial biodiversity (1), and their prevalence and prosperity are ascribed to symbiotic microorganisms associated with them (2, 3). Some symbionts are obligatory partners for their hosts due to, for example, provisioning of essential nutrients deficient in their host’s diet (4, 5), whereas other symbionts are facultative associates not necessarily needed for their host’s survival (6, 7). Such facultative symbionts often affect a variety of phenotypes of their hosts either positively or negatively, depending on environmental conditions, including manipulating the host’s reproduction (8), conferring the host’s resistance to natural enemies (9), enhancing the host’s tolerance to environmental stresses (10), and modifying the host’s food plant range (11).

Aphids (Hemiptera: Aphididae) consist of some 5,000 described species, live solely on plant phloem sap, and include a number of agricultural pest species (12). Among them, the pea aphid Acyrthosiphon pisum is the best-studied model species, and the diversity of the symbiotic bacteria and their biological functions have been investigated in detail (4, 6). Almost all aphid species, including A. pisum, are associated with the obligate bacterial symbiont Buchnera aphidicola in specialized cells called bacteriocytes. B. aphidicola exhibits 100% infection frequencies in host populations and complements the host’s nutritionally unbalanced plant sap diet by synthesizing essential amino acids (5, 13). In addition to B. aphidicola, A. pisum frequently harbors facultative bacterial symbionts such as Serratia symbiotica, Regiella insecticola, Hamiltonella defensa, *Rickettsia* sp., *Spiroplasma* sp., and others (14–18), which can have a variety of effects on the host’s phenotypes with ecological consequences, including resistance to parasitoid wasps (19), protection against pathogenic fungi (20), tolerance to elevated temperature (10), influence on food plant range (11), and skewing of sex ratios (21).

The genus *Rickettsiella* constitutes the gammaproteobacterial order *Legionellales* together with the genera *Legionella* and *Coxiella* (22). All members of the *Legionellales* are specialized for endoparasitic/symbiotic lifestyle within eukaryotic cells, and some are known as human pathogens. *Coxiella* bacteria are mostly associated with ticks, and C. burnetii is known to be the causative agent of Q fever (23, 24). Recently, it has become evident that many, if not all, *Coxiella* bacteria are either facultative or obligate endosymbiotic associates stably maintained through generations of their host ticks (25–27), and some of them exhibit conspicuous reductive genome evolution (28, 29). *Legionella* species are endocellularly associated with aquatic protozoans such as amoebas and ciliates, and Legionella pneumophila is notorious as the causative agent of Legionnaires’ disease (30, 31). *Rickettsiella* species have been reported to be pathogenic to insects, arachnids, and terrestrial crustaceans (32, 33). Members of the genus *Rickettsiella* from diverse insects and other arthropods, including *R. papillae* from beetle grubs, R. grylli from crickets, R. chironomi from midges, “*Candidatus* Rickettsiella isopodorum” (here referred to as "*Ca*. Rickettsiella isopodorum") from woodlice, and others, have been described, whereas the designations for a number of “*Candidatus* Rickettsiella” species thus far described have been phylogenetically regarded as synonyms of other species representing pathotypes of different host specificity (34–40). As for genomic information on *Rickettsiella*, draft genome sequences of R. grylli (NZ_AAQJ00000000 and MCRF00000000) and "*Ca*. Rickettsiella isopodorum" (LUKY00000000), which consist of 2, 430, and 33 contigs, respectively, have been previously deposited in the GenBank and investigated (41–43).

Recently, a novel *Rickettsiella* lineage was identified in European and American populations of A. pisum and described as “Candidatus Rickettsiella viridis” (44). “*Ca*. Rickettsiella viridis” is not pathogenic but is regarded as a facultative bacterial symbiont of the aphid host: its infection shows stable vertical transmission through host generations (45), its infection frequency ranges from 0% to 40% in natural host populations (18, 44–46), and its infection minimally affects fitness parameters of the aphid host (44, 45). Strikingly, a novel phenotypic effect, modification of the host’s body color, was discovered for “*Ca*. Rickettsiella viridis.” In natural populations of A. pisum, red and green morphs commonly coexist and are genetically controlled, with red dominant over green (47), and when experimentally infected with “*Ca*. Rickettsiella viridis,” genetically red aphids become green in color (44, 45). Considering the previous ecological studies demonstrating that ladybird beetles preferentially attack red aphids whereas parasitoid wasps tend to oviposit into green aphids (48, 49), the symbiont-induced body color change is potentially of ecologic relevance via affecting interactions with natural enemies (44, 45, 50). Additionally, it was reported that “*Ca*. Rickettsiella viridis” is protective to fungal pathogens via reducing aphid mortality and fungal sporulation (51).

The body color of A. pisum mainly consists of two groups of pigment molecules, namely, yellow-red color due to carotenoids and green-blue color due to polycyclic quinones and their glycosides, the so-called "aphins" (47, 52–54). Since the “*Ca*. Rickettsiella viridis” infection scarcely affects carotenoids but significantly increases green pigments in A. pisum, it was suggested that the symbiont may be involved in either production or induction of the green pigments (45), although it is unknown what mechanisms may underlie the symbiont-induced aphid color change. In this study, we determined and analyzed the complete genome of “*Ca*. Rickettsiella viridis,” thereby gaining insight into the molecular mechanisms involved in the symbiont-induced insect color change.

## RESULTS AND DISCUSSION

### Sequencing of “*Ca*. Rickettsiella viridis” genome.

Quality evaluation using quantitative PCR resulted in estimations indicating that the template DNA contained 3.65 µg of “*Ca*. Rickettsiella viridis” DNA (16.4%), 13.9 µg of B. aphidicola DNA (62.5%), and 4.68 µg of A. pisum DNA (21.1%). Of 4.5 Gb of Illumina raw sequence reads, 0.89 Gb of the reads were assembled into a circular chromosome of “*Ca*. Rickettsiella viridis.” Hence, about 20.0% of the reads represented the “*Ca*. Rickettsiella viridis” genome, which roughly agreed with the quantitative PCR estimate.

### General features of “*Ca*. Rickettsiella viridis” genome.

**FIG 1:**
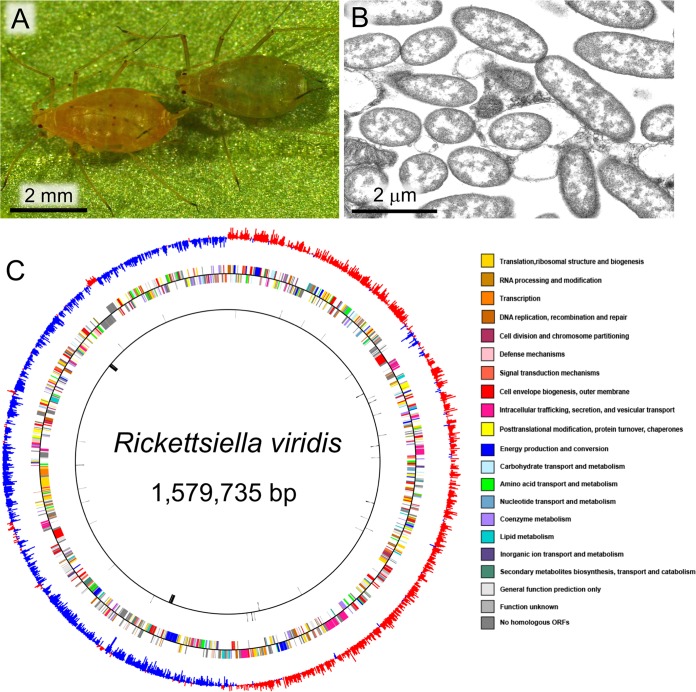
The pea aphid A. pisum and its facultative endosymbiont “*Ca*. Rickettsiella viridis.” (A) An uninfected red aphid (left) and an infected green aphid (right). (B) Transmission electron microscopy of “*Ca*. Rickettsiella viridis” cells. (C) A circular view of the “*Ca*. Rickettsiella viridis” genome. On the GC skew circle, red and blue indicate high (>0) and low (<0) (G–C)/(G+C) values (1,000 nucleotide window), respectively. On the CDS circle, colors are as indicated in the key.

The facultative bacterial symbiont “*Ca*. Rickettsiella viridis” exhibited a moderately reduced genome consisting of a circular 1,579,735-bp chromosome harboring 1,378 protein-coding open reading frames (ORFs) with an average size of 977 bp, which covered 86% of the whole genome. Of these ORFs, 963 were assigned to putative biological functions, 257 matched hypothetical proteins of unknown function, and 158 were unique to “*Ca*. Rickettsiella viridis”. Over half of the unique hypothetical proteins (92/158) were less than 100 amino acid residues in size, and it was unclear whether they represent true gene products or not. Two ribosomal operons, three small RNA genes, and 42 tRNA genes, which include at least one corresponding tRNA for each of the 20 amino acids, were identified (Fig. 1 and Table 1; see also Table S1). Besides these genes, 57 ORFs were truncated and/or interrupted by stop codons and were regarded as presumable pseudogenes (Table 1; see also Table S2). One prophage region and five insertion sequences encoding transposases were also identified on the “*Ca*. Rickettsiella viridis” genome (Table 1).

10.1128/mBio.00890-18.2 TABLE S1  Predicted genes in the “*Ca*. Rickettsiella viridis” genome. Download TABLE S1, PDF file, 0.3 MB. Copyright © 2018 Nikoh et al. 2018 Nikoh et al. This content is distributed under the terms of the Creative Commons Attribution 4.0 International license.

10.1128/mBio.00890-18.3 TABLE S2  Pseudogenes in the “*Ca*. Rickettsiella viridis” genome. Download TABLE S2, PDF file, 0.1 MB. Copyright © 2018 Nikoh et al. 2018 Nikoh et al. This content is distributed under the terms of the Creative Commons Attribution 4.0 International license.

**TABLE 1:** General genomic features of “*Ca*. Rickettsiella viridis” and allied gammaproteobacteria

Bacterium	Host and symbiotic status	Symbiotic niche(s)	Phenotypic feature(s)	Accession no.	Chromosome size (bp)	No. of plasmids	AT content (%)	Coding content (%)	No. of predicted proteins	No. of ribosomal RNAs	No. of transfer RNAs	No. of small RNA genes	No. of pseudogenes	No. of phages/ plasmid island(s)	No. of IS elements
“*Ca*. Rickettsiella viridis”	Aphid facultative symbiont	Endo-/extracellular, systemic	Aphid’s body color change	AP018005	1,579,735	0	61	86	1,378	6	42	3	57	1	5
*Rickettsiella grylli*	Cricket pathogen	Endo-/extracellular, systemic	Cricket’s pathology	AAQJ00000000^a^	1,581,239	0	62	87	1,374	6	40	3	74	0	19
"*Ca*. Rickettsiella isopodorum" RCFS	Terrestrial isopod pathogen	Endo-/extracellular, systemic	Terrestrial isopod’s pathology	LUKY00000000^b^	1,493,003	0	63	87	1,254	2	40	4	39	1	2
*Coxiella burnetii* RSA 493	Tick symbiont/ human pathogen	Endocellular, systemic	Human pathology (Q fever)	NC_002971	1,995,281	1	57	78	1,811	3	42	4	143	0	28
*Legionella pneumophila* Philadelphia 1	Amoeba symbiont/ human pathogen	Endocellular	Human pathology (legionellosis)	NC_002942	3,397,754	1	62	89	2,922	9	43	1	13	0	24
*Hamiltonella defensa* 5AT	Aphid facultative symbiont	Endo-/extracellular, systemic	Aphid’s resistance to parasitic wasp	NC_012751	2,110,331	1	60	81	2,094	9	43	2	188	5	58
*Buchnera aphidicola* APS	Aphid obligatory symbiont	Endocellular, bacteriocyte- localized	Supply of essential nutrients to aphid	NC_002528	640,681	2	74	88	562	3	32	4	14	0	0
*Escherichia coli* K-12	Human gut bacterium	Extracellular, gut cavity	Free-living gut bacterium	NC_000913	4,639,675	0	49	88	4,243	22	89	65	179	10	42

a Whole-genome shotgun sequence data, which were assembled into 2 contigs.

b Whole-genome shotgun sequence data, which were assembled into 33 contigs.

### Genomic comparison of “*Ca*. Rickettsiella viridis” with allied bacterial pathogens and symbionts.

**FIG 2:**
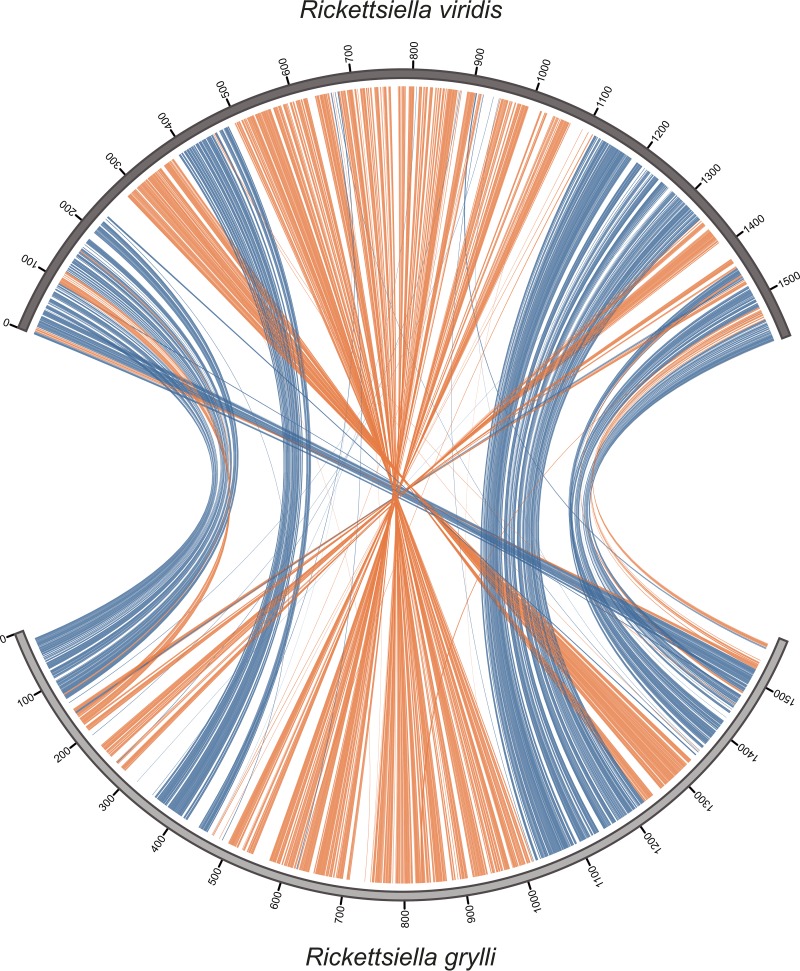
Plot of synteny between the genomes of “*Ca*. Rickettsiella viridis” and R. grylli. Orthologous genes are connected between the genomes by lines. Blue and red indicate the orthologous genes orientated in the same direction and in the reverse direction, respectively.

**FIG 3:**
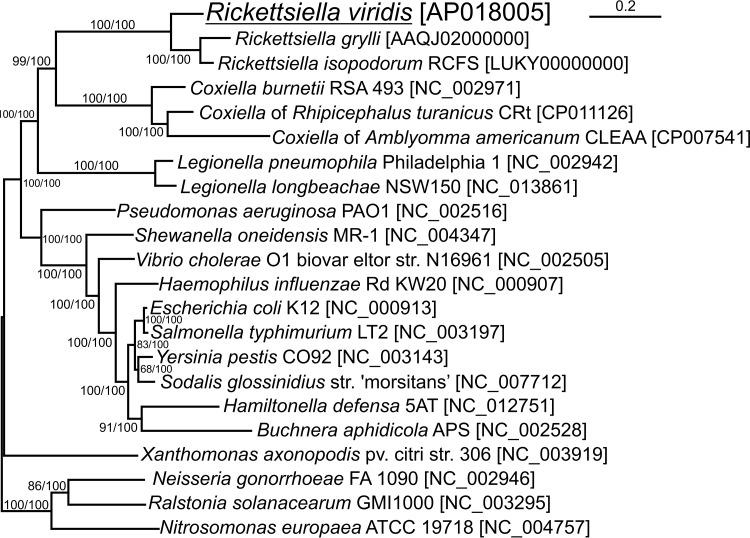
Phylogenetic placement of “*Ca*. Rickettsiella viridis” in the gammaproteobacteria. The maximum likelihood phylogeny is inferred from 53 concatenated ribosomal protein sequences (6,687 aligned amino acid sites). Statistical support values for each clade are shown at each node in the order of maximum likelihood/Bayesian analyses.

The general features of the “*Ca*. Rickettsiella viridis” genome, namely, genome size, AT content, number of ORFs, number of pseudogenes, paucity of mobile genetic elements, etc., were strikingly similar to those of Rickettsiella grylli, an endocellular pathogenic bacterium identified from crickets and grasshoppers, in contrast to those of Coxiella burnetii and Legionella pneumophila, related endocellular human pathogens belonging to the same gammaproteobacterial order, *Legionellales*, and also in contrast to those of other gammaproteobacterial endocellular aphid symbionts such as Hamiltonella defensa and Buchnera aphidicola (Table 1). The syntenic relationship of orthologous genes was well conserved between the genomes of “*Ca*. Rickettsiella viridis” and R. grylli (Fig. 2). Molecular phylogenetic analysis based on 53 ribosomal protein sequences showed close phylogenetic relationships among “*Ca*. Rickettsiella viridis,” R. grylli, and "*Ca*. Rickettsiella isopodorum" (Fig. 3). All these results consistently support the phylogenetic relationship of “*Ca*. Rickettsiella viridis” to R. grylli and "*Ca*. Rickettsiella isopodorum" belonging to the same genus, *Rickettsiella*.

**FIG 4:**
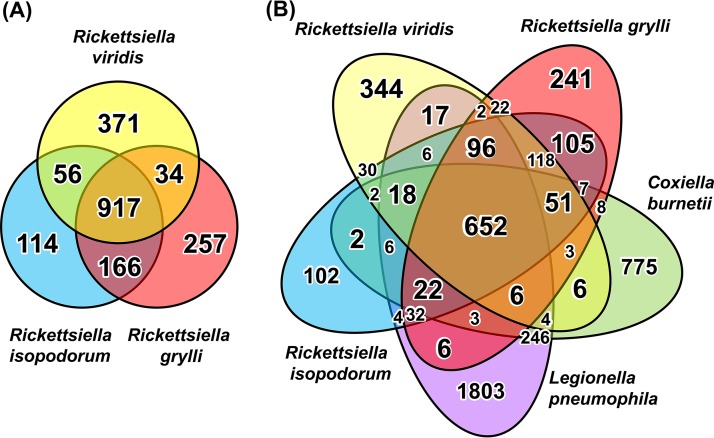
Venn diagrams comparing gene contents among “*Ca*. Rickettsiella viridis,” R. grylli, "*Ca*. Rickettsiella isopodorum," C. burnetii, and L. pneumophila. (A) “*Ca*. Rickettsiella viridis” versus *R*. grylli versus "*Ca*. Rickettsiella isopodorum." (B) “*Ca*. Rickettsiella viridis” versus *R*. grylli versus "*Ca*. Rickettsiella isopodorum" versus C. bernetii versus L. pneumophila.

Gene content analysis unveiled notable differences among the genomes of “*Ca*. Rickettsiella viridis,” R. grylli, and "*Ca*. Rickettsiella isopodorum": 371 of 1,378 protein-coding genes in the “*Ca*. Rickettsiella viridis” genome have no orthologs in the R. grylli and "*Ca*. Rickettsiella isopodorum" genomes, whereas 257 of 1,374 protein-coding genes in the R. grylli genome have no orthologs in the “*Ca*. Rickettsiella viridis” genome (Fig. 4A). Furthermore, the majority of these lineage-specific genes, namely, 344 genes in “*Ca*. Rickettsiella viridis” and 241 genes in R. grylli, have no orthologs in the genomes of C. burnetii and L. pneumophila (Fig. 4B). These patterns suggest that many lineage-specific genes evolved after the divergence of the genus *Rickettsiella*, presumably through repeated gene losses and acquisitions.

### Reductive genome evolution in “*Ca*. Rickettsiella viridis” and allied bacterial pathogens and symbionts.

The genomes of “*Ca*. Rickettsiella viridis” (1.6 Mb; an insect symbiont) and R. grylli (1.6 Mb; an insect pathogen) were smaller than the genome of C. burnetii (2.0 Mb; a tick symbiont/human pathogen) and were remarkably smaller than the genome of L. pneumophila (3.4 Mb; a protozoan symbiont/human pathogen) (Table 1). Relative-rate tests revealed that the molecular evolutionary rate of “*Ca*. Rickettsiella viridis” was comparable to that of *R. grylli*, slightly but significantly higher than that of C. burnetii, and significantly much higher than that of L. pneumophila (Table S3). These patterns suggest the possibility that reductive genome evolution has been more prominent in “*Ca*. Rickettsiella viridis,” R. grylli, and C. burnetii than in L. pneumophila, which may be relevant to their ecology and lifestyle. In their life cycle, *Rickettsiella* and *Coxiella* species are mainly associated with terrestrial arthropods, including insects, ticks, and isopods (23, 25, 33), whereas *Legionella* species are mainly associated with aquatic protozoans such as amoebas and ciliates (30, 55, 56). Considering that terrestrial extrahost conditions entailing desiccation, UV irradiation, and other environmental stresses must be extremely harsh for endocellular bacteria in contrast to aquatic extrahost conditions, horizontal transmissions across their host lineages may be relatively limited for *Rickettsiella* and *Coxiella* species in comparison with *Legionella* species. Actually, stable vertical transmission through host generations has been observed in several arthropod-associated *Rickettsiella* and *Coxiella* strains (25, 45). Although speculative, such differences in their lifestyle and transmission mode may be relevant to the different levels of their reductive genome evolution (57, 58). Notably, recent studies have unveiled that some *Coxiella* strains from ticks exhibit further genome reduction to 1.7 Mb for *Coxiella* sp. strain CRt (symbiont of Rhipicephalus turanicus) (28) and even to 0.66 Mb for *Coxiella* sp. strain CLEAA (symbiont of Amblyomma americanum) (29), which are both considered vitamin-provisioning nutritional mutualists for their host ticks (28, 29). Presumably, *Coxiella* sp. strain CLEAA may represent a more advanced stage of the host-symbiont coevolution than *Coxiella* sp. strain CRt and “*Ca*. Rickettsiella viridis.”

10.1128/mBio.00890-18.4 TABLE S3  Relative-rate test for comparing the molecular evolutionary rates of 53 concatenated ribosomal protein sequences between “*Ca*. Rickettsiella viridis” and allied endocellular bacteria of the gammaproteobacterial order *Legionellales*. Download TABLE S3, PDF file, 0.1 MB. Copyright © 2018 Nikoh et al. 2018 Nikoh et al. This content is distributed under the terms of the Creative Commons Attribution 4.0 International license.

### Metabolic capacity of “*Ca*. Rickettsiella viridis” genome.

**FIG 5:**
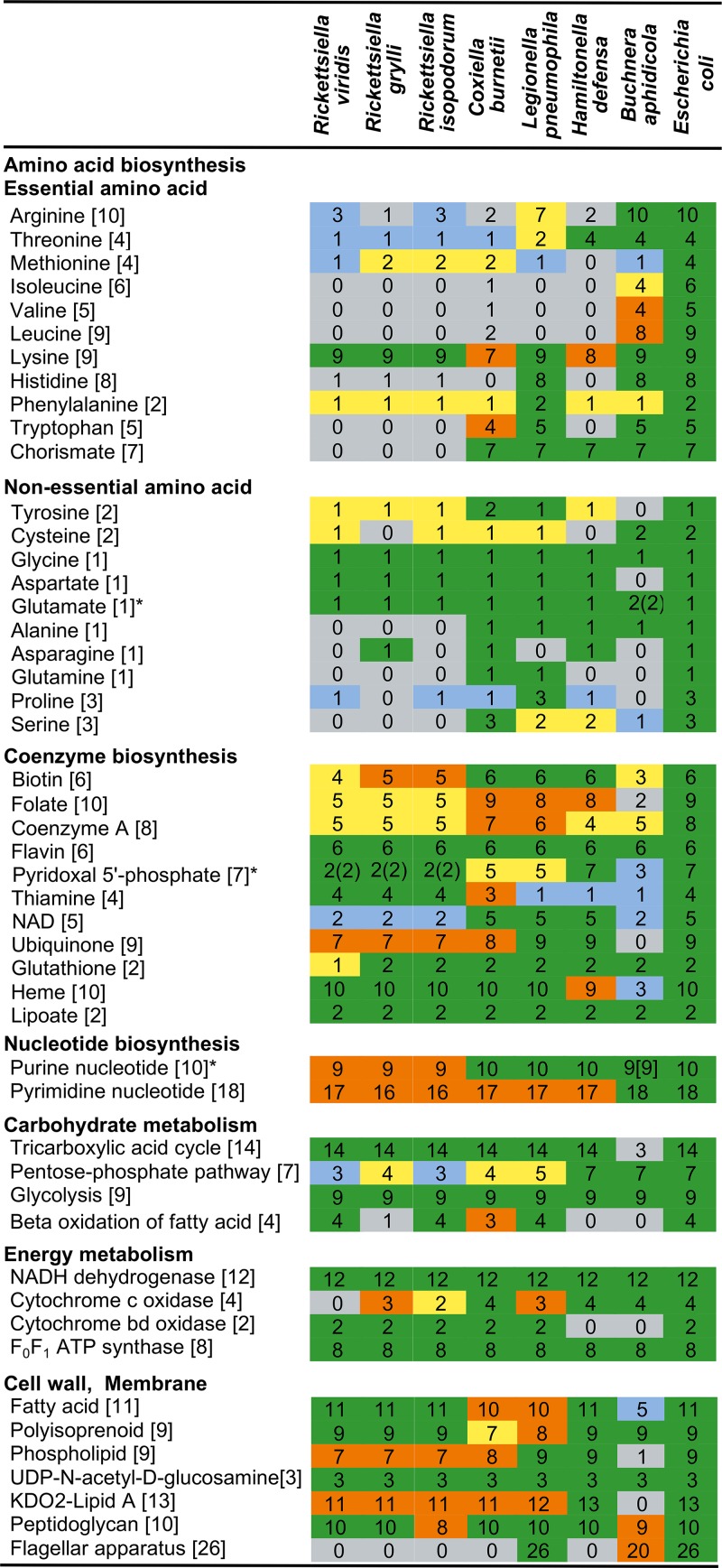
Comparison of metabolic gene repertoires among “*Ca*. Rickettsiella viridis” and allied gammaproteobacteria. The minimal number of genes for a metabolic pathway is shown in each of the brackets. Colors indicate the ratio of retained genes to the minimal gene set for a metabolic pathway as follows: green for 100%, orange for 99% to 75%, yellow for 74% to 50%, blue for 49% to 25%, and gray for 24% to 0%. Asterisks denote the presence of an alternative pathway for biosynthesis of the final product, and numbers in square brackets show the minimal number of genes for the alternative pathway.

**FIG 6:**
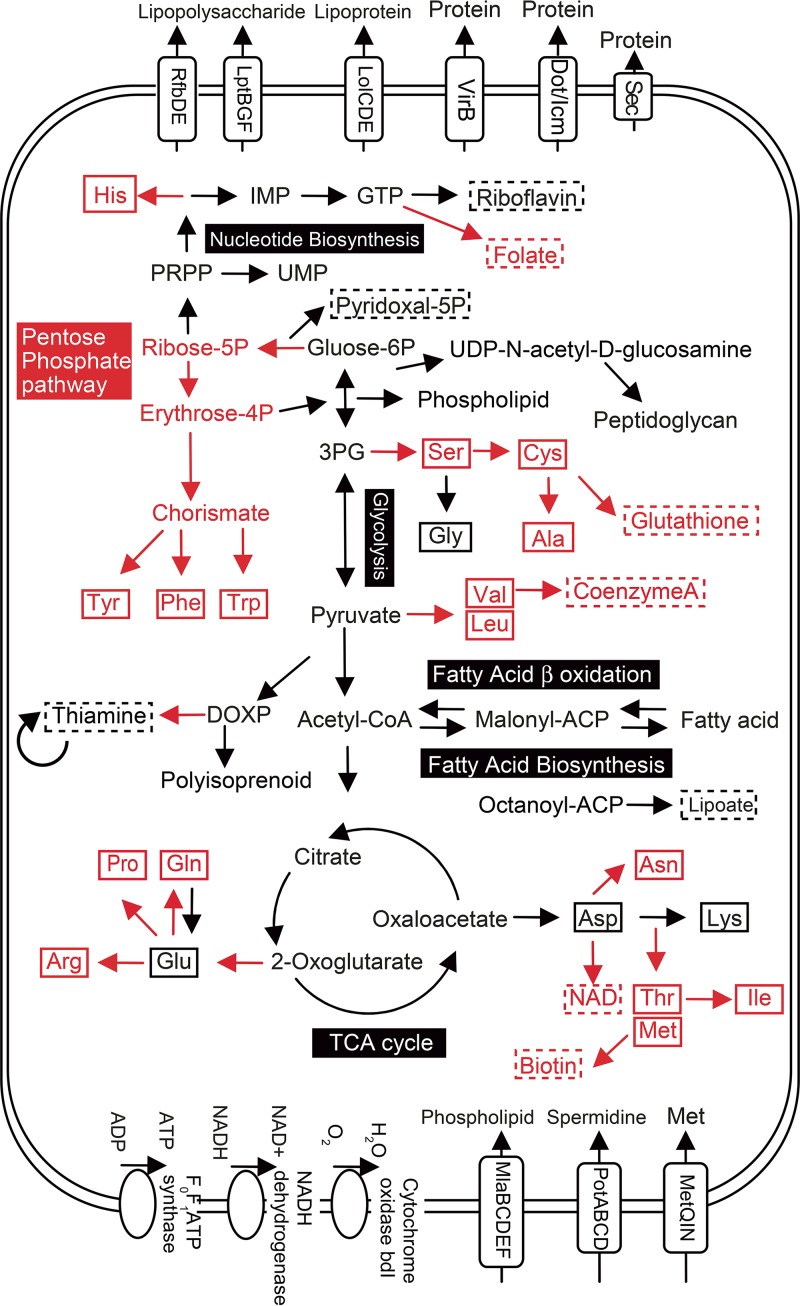
A hypothetical model of the metabolism and transport in “*Ca*. Rickettsiella viridis.” Main elements of metabolic pathways and transporters that are retained and lost in the “*Ca*. Rickettsiella viridis” genome are shown in black and red, respectively. Amino acids are in solid boxes, whereas vitamins and coenzymes are in dashed boxes. ACP, acyl carrier protein; DOXP, 1-deoxy-d-xylulose-5-phosphate pathway.

**FIG 7:**
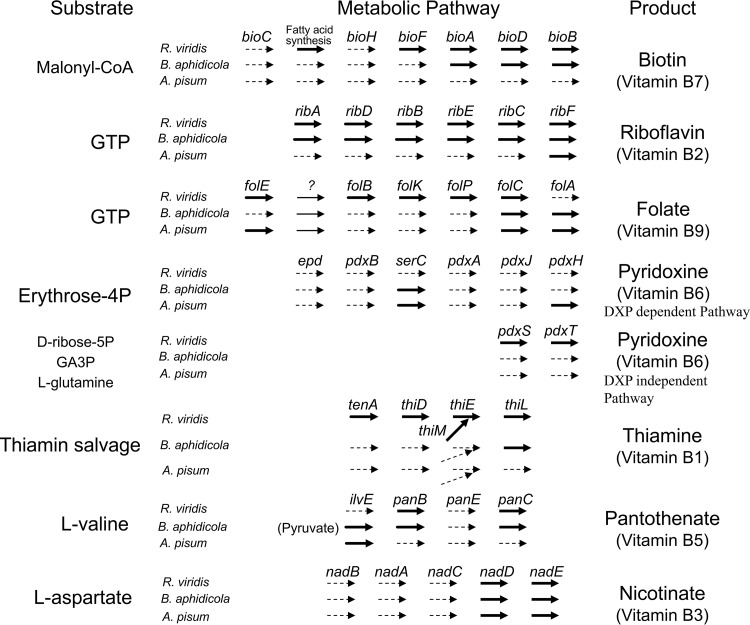
Biosynthetic pathway genes for B vitamins encoded in the genomes of “*Ca*. Rickettsiella viridis,” B. aphidicola, and A. pisum.

The “*Ca*. Rickettsiella viridis” genome, like other endocellular bacterial genomes, retained many genes responsible for basic cellular processes such as translation, replication, and energy production (Table S4). Gene content analysis revealed that “*Ca*. Rickettsiella viridis” is unable to synthesize most essential amino acids, some nonessential amino acids, and some vitamins and cofactors (Fig. 5 and 6). Probably, these molecules are provided by the host aphid and/or the coexisting primary symbiont B. aphidicola. Previous studies showed that, in some sap-sucking insects such as cicadas, spittlebugs, leafhoppers, aphids, and other hemipterans, bacteriocyte-associated cosymbionts often exhibit metabolic complementarity wherein incomplete biosynthetic pathways encoded by each of the two symbiont genomes constitute the complete metabolism as a whole when combined (59–62). However, such relationships were not observed between B. aphidicola and “*Ca*. Rickettsiella viridis” in A. pisum; for example, B vitamin synthesis genes missing in the “*Ca*. Rickettsiella viridis” genome were often also lacking in the B. aphidicola genome (Fig. 7), which should reflect the nonessential and facultative nature of “*Ca*. Rickettsiella viridis” for the host aphid (44, 45), like that of other facultative aphid symbionts, including S. symbiotica, H. defensa, and R. insecticola (63–65).

10.1128/mBio.00890-18.5 TABLE S4  Comparison of the gene repertoire between “*Ca*. Rickettsiella viridis” and allied gammaproteobacteria. Download TABLE S4, PDF file, 0.1 MB. Copyright © 2018 Nikoh et al. 2018 Nikoh et al. This content is distributed under the terms of the Creative Commons Attribution 4.0 International license.

### Absence of biosynthesis genes for polycyclic quinone pigments in “*Ca*. Rickettsiella viridis” genome.

The green color of A. pisum and other aphids has been attributed to pigment molecules mainly consisting of polycyclic quinones and/or their glucosides, the so-called aphins (45, 52–54), although the biosynthesis pathways and genes responsible for synthesis of aphins have been poorly characterized (54). Polycyclic quinones are synthesized by condensation of acetyl-coenzyme A (acetyl-CoA) and malonyl-CoA via the polyketide pathway, a process related to fatty acid synthesis (66, 67). Several types of polyketide synthase genes have been found in a variety of bacteria, including *Legionella* species (68–70). Polyketide synthases are extremely large multidomain enzymes, usually consisting of eight types of domains (71, 72). Some ORFs in the “*Ca*. Rickettsiella viridis” genome showed sequence similarity to ORFs corresponding to one of the domains of polyketide synthases. For example, *fabF* and *fabD* harbored in the “*Ca*. Rickettsiella viridis” genome exhibited partial sequence similarities to the keto synthetase domain and the acyl transferase domain of polyketide synthase gene *mscE* of Streptomyces carzinostaticus. The proteins encoded by these genes are involved in fatty acid biosynthesis. However, no ORFs in the “*Ca*. Rickettsiella viridis” genome exhibited significant sequence similarities to known polyketide synthase genes in their full lengths. These results suggest that biosynthesis genes for green polycyclic quinone pigments are not harbored in the “*Ca*. Rickettsiella viridis” genome but are likely harbored in the host aphid genome.

### Putative biological role of fatty acid catabolism genes in “*Ca*. Rickettsiella viridis” genome.

In the “*Ca*. Rickettsiella viridis” genome, we identified genes for beta-oxidation of fatty acids, which generate acetyl-CoA and recruit it into the tricarboxylic acid (TCA) cycle for ATP production (Fig. 5 and 6). Here it is notable that other endosymbiotic bacteria of A. pisum whose genomes have been determined, namely, B. aphidicola, S. symbiotica, H. defensa, and R. insecticola, possess no beta-oxidation pathway (13, 63–65), which may be relevant to the fact that the sole food of the aphid, plant phloem sap, is carbohydrate-rich but devoid of lipids and proteins (5). In this context, the function of the beta-oxidation pathway of “*Ca*. Rickettsiella viridis” may be not only for its own ATP synthesis but also for provisioning acetyl-CoA to the host. Considering that malonyl-CoA or methylmalonyl-CoA is used for synthesizing the carbon backbone of polyketides (73) and that acetyl-CoA carboxylase encoded in the “*Ca*. Rickettsiella viridis” genome catalyzes the conversion of acetyl-CoA into malonyl-CoA (Fig. 6), it seems plausible, although speculative, that the beta-oxidation capability of “*Ca*. Rickettsiella viridis” might be involved in provisioning of the substrates for the host’s synthesis of the green pigments.

### Secretion systems encoded by the “*Ca*. Rickettsiella viridis” genome.

Secretion of macromolecules across the cell envelope is important for establishing bacterial infection and virulence, and a variety of specialized molecular machineries, the so-called secretion systems, are operating in bacteria and have been classified into several evolutionarily and functionally related groups, such as the type I, II, III, IV, and V secretion systems (74). Among these, the secretion machineries ancestrally related to the bacterial conjugation systems are called the type IV secretion systems (75). The type IV secretion systems are membrane-associated transporter complexes that function to deliver DNA and protein substrates across the bacterial cell envelope to other bacterial cells, to eukaryotic host cells, or to extracellular milieu (76). A survey of the “*Ca*. Rickettsiella viridis” genome revealed several secretion systems, one of which was the Dot/Icm type IV secretion system commonly present in the endocellular bacterial members of the *Legionellales*, including R. grylli, "*Ca*. Rickettsiella isopodorum," C. burnetii, and L. pneumophila (Table S5) (77). Of 25 genes encoding the major components of the secretion system, 21 were found in the “*Ca*. Rickettsiella viridis” genome, whose orthologs were mostly conserved in the genomes of R. grylli, "*Ca*. Rickettsiella isopodorum," C. burnetii, and L. pneumophila (Table S5). In addition, the “*Ca*. Rickettsiella viridis” genome contained the VirB type IV secretion system. Of 12 genes comprising the major components of the secretion system, 9 intact genes and a pseudogene were present in the “*Ca*. Rickettsiella viridis” genome (Table S5), wherein the genes were found in two different operon-like structures. Each of the operons was located in a mobile genetic element-like region with a transposase gene and an integrase gene (see Fig. S1 in the supplemental material), suggesting the possibility that these operons may potentially be transposable. While R. grylli and C. burnetii were devoid of the VirB type IV secretion system, L. pneumophila possessed 10 genes comprising the secretion system (Table S5).

10.1128/mBio.00890-18.1 FIG S1  Gene orders of VirB type IV secretory systems harbored in mobile genetic element-like regions in the genome of “*Ca*. Rickettsiella viridis.” Pentagons indicate genes, with the coding direction to the tip indicated. Pentagons encircled by a dashed line represent pseudogenes. Red pentagons represent *virB* genes. Gray pentagons represent genes encoding proteins of unknown function. The nucleotide sequences of these two regions are exactly the same. Download FIG S1, PDF file, 0.1 MB. Copyright © 2018 Nikoh et al. 2018 Nikoh et al. This content is distributed under the terms of the Creative Commons Attribution 4.0 International license.

10.1128/mBio.00890-18.6 TABLE S5  Comparison of secretion systems encoded in the genomes of “*Ca*. Rickettsiella viridis” and allied gammaproteobacteria. Download TABLE S5, PDF file, 0.1 MB. Copyright © 2018 Nikoh et al. 2018 Nikoh et al. This content is distributed under the terms of the Creative Commons Attribution 4.0 International license.

### Effector proteins for Dot/Icm type IV secretion system.

Thus far, a number of studies have extensively screened for L. pneumophila genes encoding effector proteins secreted by the Dot/Icm type IV secretion system and have identified some 300 confirmed or presumable effector protein-encoding genes in the bacterial genome, although the biological functions are still unknown for the majority of them (78, 79). In the “*Ca*. Rickettsiella viridis” genome, we identified 10 genes, *arp*/*ankH*/*sdcA*, *legK1/legK3*, *lepB*, L. pneumophila g2359 (*lpg2359*), *lpg2628*, *mavN*, *ravC*, *ravJ*, *rsmE*, and *sidP*, that are orthologous to the effector genes identified in L. pneumophila (Table S6). In L. pneumophila, *sidP* contains a CX_5_R motif, encodes a phosphoinositide-3-phosphatase, and presumably affects the host’s lysosomal trafficking by delaying the phosphoinositide cascade (80); *legK1*, one of five L. pneumophila genes with eukaryotic protein kinase motifs, is suggested to be involved in modulation of macrophage defense or inflammatory responses via activating the host NF-κB signaling (81); *ankH* encodes an effector protein with two ankyrin motifs and is needed for intracellular proliferation of L. pneumophila (82); and *mavN* encodes a putative transmembrane protein, targets vacuolar membrane, and facilitates intravacuolar iron acquisition by L. pneumophila (83, 84). It seems likely, although speculative, that these effector orthologs of “*Ca*. Rickettsiella viridis” may be similarly translocated via the Dot/Icm type IV secretion system and involved in infection processes and cytological modifications in the aphid-*Rickettsiella* association. The list of the effector gene candidates detected by a homology-based survey of the “*Ca*. Rickettsiella viridis” genome (Table S6) is no doubt a conservative estimate, and many more effectors are to be identified in future studies. It should also be noted that there may be additional effector molecules related to the VirB type IV secretion system encoded in the “*Ca*. Rickettsiella viridis” genome (see Table S5).

10.1128/mBio.00890-18.7 TABLE S6  Genes of “*Ca*. Rickettsiella viridis” encoding putative effector proteins, which are orthologous to effector protein genes identified in L. pneumophila. Download TABLE S6, PDF file, 0.1 MB. Copyright © 2018 Nikoh et al. 2018 Nikoh et al. This content is distributed under the terms of the Creative Commons Attribution 4.0 International license.

### “*Ca*. Rickettsiella viridis” proteins with eukaryotic motifs.

As depicted above (see Table S6), the effector proteins of “*Ca*. Rickettsiella viridis,” and also those of L. pneumophila, often contain eukaryotic protein motifs and functional domains (85, 86). Hence, in an attempt to identify more candidate effector protein genes in “*Ca*. Rickettsiella viridis,” we surveyed the “*Ca*. Rickettsiella viridis” genome using SMART (87), thereby retrieving a total of 31 protein-coding genes with eukaryotic motifs: 16 genes with ankyrin repeats; 6 genes with coiled coils; 3 genes with a serine/threonine kinase motif; and 6 genes with a GMP reductase motif, RCC1 repeats, tetratricopeptide repeats, an AAA motif, a Ras motif, or an orotidine-5′-phosphate decarboxylase motif (Table S7). Although biological aspects of these genes are unknown, it is possible that they represent symbiont-produced effector molecules functioning in the association between the aphid and “*Ca*. Rickettsiella viridis.”

10.1128/mBio.00890-18.8 TABLE S7  Genes of “*Ca*. Rickettsiella viridis” encoding proteins with eukaryotic motifs. Download TABLE S7, PDF file, 0.1 MB. Copyright © 2018 Nikoh et al. 2018 Nikoh et al. This content is distributed under the terms of the Creative Commons Attribution 4.0 International license.

### Conclusion and perspective.

The genome of “*Ca*. Rickettsiella viridis” determined in this study, which is the first completely determined genome of a *Rickettsiella* species, illuminates the nature of the bacterium as a moderately genome-reduced, facultative endosymbiont of the aphid A. pisum which is phylogenetically allied to entomopathogenic *Rickettsiella* species. The gene repertoire analyses strongly suggest that “*Ca*. Rickettsiella viridis” is involved in the symbiosis-induced body color change indirectly, in which the symbiont itself cannot produce the green pigments in the absence of relevant synthetic genes but probably activates the host’s pathways for synthesis of the green pigments. Future studies should focus on transcriptomic and functional analyses of the genes involved in synthesis of the green pigments encoded in the A. pisum genome (88), in which polyketide synthesis genes and fatty acid synthesis genes are likely candidates.

Not only the mechanisms of the green pigment synthesis but also the mechanisms as to how “*Ca*. Rickettsiella viridis” activates the host’s biosynthesis pathway for the green pigments are of profound interest. In the “*Ca*. Rickettsiella viridis” genome, we identified a number of type IV secretion system genes and possible effector protein genes. These molecular machineries may function for infection and proliferation of the symbiont in the host insect cells. It seems likely, although speculative, that the molecular machineries may also be involved in manipulation of the host’s cellular processes and metabolic pathways, thereby realizing the upregulation of the host’s green pigment production. Judging from the comparative genomics of *Coxiella* and *Legionella* (and *Rickettsiella* in this study), members of the gammaproteobacterial order *Legionellales* must have ancestrally acquired the molecular mechanisms consisting of the Dot/Icm type IV secretion system (77), which has been followed by the evolution of extremely diverse effector proteins for establishing their endocellular lifestyle within eukaryotic host cells (89). The presence of these molecular machineries may have predisposed the ancestor of “*Ca*. Rickettsiella viridis” to achieve the striking symbiotic phenotype of modifying the host’s body color.

For a long time, C. burnetii and L. pneumophila have been recognized as human pathogens that cause Q fever and Legionnaires’ disease, respectively (24, 30). However, recent ecological studies have revealed that these human pathogens are rather exceptional among the diversity of *Coxiella* and *Legionella* species; the majority of them exist in the environment as endocellular bacteria in ticks for *Coxiella* species (25, 26) and as endocellular bacteria in aquatic amoebas and ciliates for *Legionella* species (30, 31). *Rickettsiella* species are known as endocellular pathogens/symbionts of insects and other arthropods without known vertebrate hosts (32, 33). The genomic commonalities and differences observed in the members of *Legionellales* highlight the molecular, cellular, and evolutionary continuum across pathology, virulence, manipulation, and other physiological and ecological consequences of parasitic/symbiotic associations between the endocellular bacteria and their eukaryotic hosts.

## MATERIALS AND METHODS

### Insect and DNA preparation and histology.

We used a laboratory A. pisum strain, 4TV^amp/RA04acg^, which was generated by artificial transfer of “*Ca*. Rickettsiella viridis” from donor aphid strain RA04^acg^ into recipient aphid strain 4 TV^amp^ (45). These aphid strains were originally collected in locations around Rennes, France, from the red clover Trifolium pratense and the alfalfa Medicago sativa, respectively, and the experimental aphid strains have been maintained in the laboratory on seedlings of the broad bean Vicia faba (45). In total, 48 adult female aphids were dissected in phosphate-buffered saline. Debris was removed through the use of 100-, 50-, 11-, and 5-µm-pore-size filters, and the filtrate was subjected to DNA extraction. Aliquots of the DNA sample were subjected to quantitative PCR to estimate the relative DNA contents of “*Ca*. Rickettsiella viridis,” B. aphidicola, and A. pisum by targeting the following genes: the *gyrB* gene of “*Ca*. Rickettsiella viridis” using primers RclGyrB-AF1 and RclGyrB-AR1 (45): the *dnaK* gene of B. aphidicola using primers BuchDnaK-AF1 and BuchDnaK-AR1 (90); and the elongation factor 1α gene of A. pisum using primers ApisEF-422F and ApisEF-522R (91). The DNA amount of the each target organism was calculated as *M* = *Γ* × *N* × *M*_Bp_ × *N_A_*^−1^, where *Γ* is the genome size (600 Mb for A. pisum [88], 0.64 Mb for B. aphidicola [13], and, tentatively, 1.5 Mb for “*Ca*. Rickettsiella viridis”), *N* is the copy number of the target sequence (determined by real-time PCR), *M*_Bp_ is the mean molar mass of a base pair (660 g mol^−1^), and *N*_*A*_ is Avogadro’s number (6.02 × 10^23^ mol^−1^). For transmission electron microscopy, dissected aphid tissues were prefixed with glutaraldehyde, postfixed with osmium tetroxide, embedded in epoxy resin, processed into ultrathin sections, stained with uranyl acetate and lead citrate, and observed under a transmission electron microscope as previously described (45).

### Genome analysis.

Using the genome DNA extracted as described above, a genomic library was constructed with an S220 Focused-Ultrasonicator (Covaris, Woburn, MA, USA) for fragmentation, Pippin Prep (Sage Science, Beverly, MA, USA) for size selection (target fragment size = 200 to 400 bp), and a TruSeq DNA Sample Prep kit (Illumina, CA, USA) for adapter ligation and library amplification. The library was sequenced (101 bp from each end) on a HiSeq 2000 platform (Illumina). A total of 44,139,360 raw reads (4.5 Gb) was obtained from the library. The obtained reads were assembled after an *in silico* elimination of contaminated sequences derived from the primary symbiont B. aphidicola. After filtering low-quality and adapter sequences, we mapped the reads to the genomes of B. aphidicola strains A5 (NC_011833) and APS (NC_002528) using bowtie2 (ver. 2.0.0) and used the unmapped 0.89 Gb of sequences for Velvet (ver.1.2.07) assembling. After several rounds of optimization of the Velvet parameters, we obtained the best assembly with k-mer = 91 and exp_cov = 30. The resultant assembly comprised 149 scaffolds (>1,000 bp) containing 1,865 kbp, among which 4 scaffolds (1,554 kbp, in total) were identified as representing the “*Ca*. Rickettsiella viridis” genome on the basis of high similarity to the genome of R. grylli (NZ_AAQJ02000000). The gaps among the scaffolds were closed by Sanger sequencing. Putative protein-coding sequences (CDSs) were predicted using Glimmer3.0 (92). The annotation of CDSs was based on results of BLASTP searches against UniProt and the NCBI nonredundant protein database. Table S1 lists the annotated CDSs. The CDSs that exhibited database matches to a functional gene of other bacteria but were interrupted by frame shifts and/or stop codons were regarded as pseudogenes. To find pseudogenes in spacer regions between CDSs, BLASTX searches were conducted against UniProt using the spacer sequences as the queries. Table S2 lists the identified pseudogenes. tRNA genes were predicted by tRNAscan-SE (93). Other noncoding RNAs were identified in accordance with their similarity to Escherichia coli homologues. The bacterial insertion sequences were predicted using IS finder (94).

### Molecular phylogenetic and evolutionary analyses.

A set of 53 ribosomal protein genes for which orthologs were commonly identified in “*Ca*. Rickettsiella viridis” and other related representatives of the family *Legionellales* was selected for phylogenetic analyses (Table S1). Each of the ortholog sets was aligned using MAFFT 5.6 (95), and all the alignments were concatenated. Alignment gaps and ambiguously aligned sites were excluded from analysis. The Le and Gascuel (LG) gamma-distributed rate (+G) invariable sites (+I) substitution model for the amino acid sequences was selected under the Akaike criterion using ProtTest v3.4.2 (96). Molecular phylogenetic analyses were conducted by two methods: maximum likelihood analysis using RAxML version 8.2.0 (97) and Bayesian analysis using MrBayes 3.1.2 (98). Bootstrap values for maximum likelihood phylogeny were obtained by 1,000 resamplings. Posterior probabilities were estimated for Bayesian phylogeny. A relative-rate test was performed using RRTree (99) on the basis of amino acid distances calculated from the concatenated alignment of the 53 ribosomal protein sequences.

### Accession number(s).

The annotated genome sequence of “*Ca*. Rickettsiella viridis” has been deposited in the DNA Data Bank of Japan with the accession number AP018005.
