# Supplementary material for: Genomic Insight into Symbiosis-Induced Insect Color Change by a Facultative Bacterial Endosymbiont, “Candidatus Rickettsiella viridis”
Source: mBio. 2018 Jun 12;9(3):e00890-18. doi: 10.1128/mBio.00890-18 (PMC6016236; doi:10.1128/mBio.00890-18)
Supplement: TABLE S1 [file mbo003183938st1.pdf]

TABLE S1 Predicted genes in the "*Ca.* Rickettsiella viridis" genome.

	Location	Strand	Nucleotide length	Gene	COG category[1]	Products	Phylogentic analysis & relative rate test[2]
Proteins							
	74..1423	+	1350	*dnaA*	L	Chromosomal replication initiator protein DnaA	
	1433..2581	+	1149	*dnaN*	L	DNA polymerase III subunit beta	
	2588..3673	+	1086	*recF*	L	DNA replication and repair protein RecF	
	3743..4000	+	258	*sugE*	V	Small multidrug resistance protein	
	4040..4318	-	279			hypothetical protein	
	4366..5106	-	741		R	Rhodanese-related sulfurtransferase	
	5312..6685	+	1374	*glyQS*	J	Glycine--tRNA ligase	
	6707..7402	-	696		R	Alanine racemase domain protein	
	7473..10067	+	2595	*acnB*	C	Aconitate hydratase 2	
	10476..10781	+	306		T	Uncharacterized protein	
	10797..11516	-	720	*fabG*	I	Short-chain dehydrogenase/reductase SDR	
	11638..12510	-	873		K	Transcriptional regulator LysR family	
	12798..13295	+	498			hypothetical protein	
	13547..14536	-	990	*birA*	H	Biotin/acetyl-CoA-carboxylase ligase	
	14556..15167	-	612			Putative uncharacterized protein	
	15655..17163	-	1509	*comM*	O	ComM-like protein	
	17239..17511	-	273		S	Putative uncharacterized protein	
	17535..19097	-	1563		R	Aminoglycoside phosphotransferase family enzyme	
	19230..20372	+	1143	*cfa*	I	Cyclopropane fatty acyl phospholipid synthase	
	20554..21093	+	540	*rppH*	V	RNA pyrophosphohydrolase	
	21272..23545	+	2274	*ptsP*	T	Phosphoenolpyruvate protein phosphotransferase PtsP	
	23556..24902	+	1347		C	FAD linked oxidase domain protein	
	24977..25342	-	366			hypothetical protein	
	25401..27476	-	2076	*prlC*	E	Oligopeptidase A	
	27559..28038	-	480	*recX*	O	Regulatory protein RecX	
	28163..29203	-	1041	*recA*	L	recombinase A	
	29296..30732	+	1437	*hldE*	M	Bifunctional protein HldE	
	30729..31682	+	954	*hldD*	M	ADP-L-glycero-D-manno-heptose-6-epimerase	
	31754..32095	+	342		L	DNA uptake protein	
	32155..33198	-	1044	*fbaB*	G	Fructose-bisphosphate aldolase	
	33507..35402	+	1896			Hypothetical membrane spanning protein	
	35616..35969	+	354		C	ATP synthase I chain	
	35966..36766	+	801	*atpB*	C	ATP synthase subunit a	
	36798..37103	+	306	*atpE*	C	ATP synthase C chain	
	37131..37604	+	474	*atpF*	C	ATP synthase subunit b	
	37683..38267	+	585	*atpH*	C	ATP synthase subunit delta	
	38284..39828	+	1545	*atpA*	C	ATP synthase subunit alpha	
	39835..40695	+	861	*atpG*	C	ATP synthase gamma chain	
	40758..42146	+	1389	*atpD*	C	ATP synthase subunit beta	
	42160..42591	+	432	*atpC*	C	ATP synthase epsilon chain	
	42670..44061	+	1392	*glmU*	M	Bifunctional protein GlmU	
	44096..44488	-	393			hypothetical protein	
	44573..45340	-	768	*ampD*	M	N-acetylmuramoyl-L-alanine amidase AmiD	
	45349..45804	-	456		M	Protein required for attachment to host cells	
	45853..46515	-	663	*rpiA*	G	Ribose-5-phosphate isomerase A	
	46635..46844	-	210			hypothetical protein	
	47595..48320	+	726	*dsbA*	O	Thiol:disulfide interchange protein	
	48321..49112	+	792	*mlaF*	M	ABC-type transporter Mla maintaining outer membrane lipid asymmetry ATPase component MlaF	
	49116..49904	+	789	*mlaE*	M	ABC-type transporter Mla maintaining outer membrane lipid asymmetry permease component MlaE	
	49905..50375	+	471	*mlaD*	M	ABC-type transporter Mla maintaining outer membrane lipid asymmetry periplasmic component MlaD	
	50372..50974	+	603	*mlaC*	M	ABC-type transporter Mla maintaining outer membrane lipid asymmetry periplasmic MlaC component	
	50992..51282	+	291	*mlaB*	M	ABC-type transporter Mla maintaining outer membrane lipid asymmetry MlaB component	
	51295..52572	+	1278	*murA*	M	UDP-N-acetylglucosamine 1-carboxyvinyltransferase	
	52737..53471	-	735	*pqqC*	H	Coenzyme PQQ synthesis protein C	
	53758..54276	+	519			hypothetical protein	
	54345..56123	-	1779		U	Hemolysin activation/secretion protein	
	56214..56849	-	636	*eda*	G	2-dehydro-3-deoxyphosphogluconate aldolase/4-hydroxy-2-oxoglutarate aldolase	
	56833..57030	-	198			hypothetical protein	
	57091..58029	+	939	*kdgK*	G	2-dehydro-3-deoxygluconokinase	
	58031..59074	+	1044	*mro*	G	Aldose 1-epimerase	
	59112..59951	+	840		G	Gluconolactonase	
	60184..61551	+	1368	*ywtG*	G	D-xylose-proton symporter	
	61544..62389	+	846		I	Short chain dehydrogenase	

62444..63652	+	1209	*rspA*	M	Starvation sensing protein
63698..65722	-	2025	*htpG*	O	Chaperone protein HtpG
65858..66259	-	402			hypothetical protein
66423..67184	-	762		R	Uncharacterized protein
67211..67840	-	630		S	Uncharacterized protein
67863..68540	-	678		K	Peptidase S24-like domain protein
68795..69202	+	408		T	GacS/BarA family sensor protein
69447..69617	-	171			hypothetical protein
69872..71080	-	1209	*tyrP*	E	tryptophan/tyrosine permease
71511..71903	+	393	*sufA*	O	Iron-sulfur cluster assembly protein
71916..72185	+	270		G	Phosphocarrier protein HPr
72189..73100	+	912		M	Uncharacterized protein
73227..74588	-	1362	*pmbA*	R	peptidase U62 modulator of DNA gyrase
74700..75125	+	426	*yicC*	P	Rhodanese domain protein
75149..75415	+	267	*grxC*	O	Glutaredoxin 3
75412..75921	+	510	*secB*	U	Protein-export protein SecB
75928..76611	+	684	*coaE*	H	Dephospho-CoA kinase
76740..78989	+	2250			putative membrane protein
79095..79922	-	828			hypothetical protein
80274..81638	-	1365	*tolC*	U	Putative type I secretion outer membrane efflux TolC [illegible]
81731..82354	+	624	*nudF*	V	ADP-ribose pyrophosphatase
82357..84258	+	1902	*parE*	L	DNA topoisomerase 4 subunit B
84313..85179	-	867			hypothetical protein
85412..86188	+	777		C	Putative L-lactate dehydrogenase
86166..86828	-	663		R	Caffeoyl-CoA O-methyltransferase
87062..87214	-	153			hypothetical protein
87230..87847	+	618	*cynT*	P	Carbonic anhydrase
87874..88608	-	735		H	cyclopropane fatty acyl phospholipid synthase
88652..89242	-	591	*rnfB*	C	Predicted NADH:ubiquinone oxidoreductase- subunit RnfB
89239..90954	-	1716	*metG*	J	Methionine--tRNA ligase
90944..91063	-	120			hypothetical protein
91243..92064	+	822	*mrp*	D	ATPase-like ParA/MinD
92139..92705	+	567	*dcd*	F	Deoxycytidine triphosphate deaminase
92717..94657	-	1941			Uncharacterized protein
94810..95601	-	792	*rsmA*	J	Ribosomal RNA small subunit methyltransferase A
95618..96766	-	1149	*pdhC*	C	Branched-chain alpha-keto acid dehydrogenase subunit E2
96763..97743	-	981	*pdhB*	C	Transketolase
97740..98825	-	1086	*pdhA*	C	Pyruvate dehydrogenase E1 component alpha subunit
98831..99898	-	1068		E	Glu/Leu/Phe/Val dehydrogenase
100103..101278	-	1176	*cca*	J	Multifunctional CCA protein
101263..101415	-	153			hypothetical protein
101455..103635	-	2181			hypothetical protein
103743..105665	-	1923			Lipoprotein
107519..108403	+	885	*psd*	I	Phosphatidylserine decarboxylase proenzyme
108476..108691	+	216			hypothetical protein
108782..110113	+	1332		R	FOG: TPR repeat
110302..110796	+	495		I	Acyl-CoA hydrolase
111014..111790	+	777	*gloB*	R	Hydroxyacylglutathione hydrolase
112082..113242	+	1161			Uncharacterized protein
113383..113748	+	366	*folB*	H	Dihydroneopterin aldolase
113845..116433	+	2589	*mutS*	L	DNA mismatch repair protein MutS
116430..117287	+	858	*pssA*	I	CDP-diacylglycerol--serine O-phosphatidyltransferase
117369..118466	-	1098	*trpS*	J	Tryptophanyl-tRNA synthetase
118429..118578	+	150			hypothetical protein
118656..119540	+	885			hypothetical protein
119614..121185	+	1572	*pckA*	C	Phosphoenolpyruvate carboxykinase [ATP]
121244..122101	-	858	*proC*	E	Pyrroline-5-carboxylate reductase
122117..122794	-	678		M	Mannose-1-phosphate guanyltransferase
122799..123797	-	999		R	Aminoglycoside phosphotransferase
123993..124880	+	888	*dapA*	E	4-hydroxy-tetrahydrodipicolinate synthase
124964..125077	-	114			hypothetical protein
126267..127646	+	1380	*qxtA*	C	Cytochrome bd-I oxidase subunit I
127647..128642	+	996	*qxtB*	C	Cytochrome d ubiquinol oxidase subunit II
128833..129909	-	1077			hypothetical protein
130125..130634	+	510	*def*	J	Peptide deformylase
130631..131578	+	948	*fmt*	J	Methionyl-tRNA formyltransferase
131571..132872	+	1302	*sun*	J	Ribosomal RNA small subunit methyltransferase B
132905..133438	+	534	*yiaD*	M	OmpA/MotB domain protein
133530..134381	-	852			Uncharacterized protein
134600..136360	+	1761	*argS*	J	Arginine--tRNA ligase
136360..137013	+	654		D	Putative uncharacterized protein
137126..137683	+	558	*hslV*	O	ATP-dependent protease subunit HslV
137711..139204	+	1494	*hslU*	O	ATP-dependent protease ATPase subunit HslU
139208..139570	+	363		S	DUF971 domain containing protein
139600..139884	-	285			Uncharacterized protein

139946..141163	-	1218	*tyrS*	J	Tyrosine--tRNA ligase	
141153..142010	-	858	*rsmI*	J	Ribosomal RNA small subunit methyltransferase I	
142091..143452	+	1362		E	Arginine/agmatine antiporter	
143462..146461	+	3000		M	small-conductance mechanosensitive channel	
146543..147661	+	1119	*murG*	M	UDP-N-acetylglucosamine--N-acetylmuramyl-(pentapeptide) pyrophosphoryl-undecaprenol N-acetylglucosamine transferase	
147679..148353	-	675			Putative T7 phage protein	
148397..150820	-	2424		U	putative periplasmic protein	
150817..152553	-	1737	*yrdA*	M	Surface antigen (D15)	
152714..153850	-	1137			Phosphate transporter	
154056..155270	+	1215	*dacC*	M	D-alanyl-D-alanine carboxypeptidase	
155363..155476	+	114			hypothetical protein	
155476..156366	+	891		I	Putative 1-acyl-sn-glycerol-3-phosphate acyltransferase	
156436..157374	+	939	*hemC*	H	Porphobilinogen deaminase	
157360..159312	-	1953			hypothetical protein	
159575..160261	-	687	*nth*	L	Endonuclease III	
160218..160865	-	648		K	Peptidase S24-like domain protein	
161077..161334	+	258	*rpsO*	J	30S ribosomal protein S15	x
161433..163568	+	2136	*pnp*	J	Polyribonucleotide nucleotidyltransferase	
163636..164061	+	426	*pspE*	P	Rhodanese domain protein	
164615..165769	+	1155			Uncharacterized protein	
165945..167021	+	1077	*pyrD*	F	Dihydroorotate dehydrogenase	
167021..168073	+	1053	*carA*	E	Carbamoyl-phosphate synthase	
168081..171272	+	3192	*carB*	E	Carbamoyl-phosphate synthase	
171291..172214	+	924	*pyrB*	F	Aspartate carbamoyltransferase	
172426..174609	-	2184			BRCT domain protein	
175026..176252	+	1227	*pyrC*	F	Dihydroorotase	
176370..177281	-	912	*hemY*	S	HemY domain protein	
177278..178279	-	1002	*hemX*	S	uroporphyrin-III C-methyltransferase	
178240..179043	-	804	*hemD*	H	Uroporphyrinogen-III synthase	
179154..180335	+	1182	*pgk*	G	Phosphoglycerate kinase	
180346..181776	+	1431	*pyk*	G	Pyruvate kinase	
181786..182211	-	426	*fur*	P	Ferric uptake regulation protein	
182300..182596	+	297	*bamE*	M	Outer membrane protein assembly factor BamE	
182613..182900	-	288	*rnfH*	V	protein RnfH	
182910..183350	-	441		J	Cyclase/dehydrase	
183429..183905	+	477	*smpB*	O	SsrA-binding protein	
184560..185786	+	1227		X	Integrase family protein	
185888..186772	+	885		V	Aminoglycoside/hydroxyurea antibiotic resistance kinase	
186880..187467	+	588	*yvdD*	R	Putative phosphoribohydrolase	
187512..188378	+	867		K	two-component sensor histidine kinase	
188335..189624	+	1290		G	Major facilitator family transporter	
189884..190807	+	924			hypothetical protein	
190898..191884	+	987		T	Putative sensory box sensor histidine kinase/response regulator	
192190..192723	+	534		R	Complete genome; segment 4/17	
192839..193324	+	486			Terminase small subunit	
193699..194289	-	591			hypothetical protein	
195116..196039	+	924		X	Putative RNA-directed DNA polymerase (Reverse transcriptase)	
196172..196426	+	255			hypothetical protein	
196654..197172	+	519	*gcvR*	E	Amino acid-binding ACT domain protein	
197209..197670	+	462	*bcp*	O	Putative peroxiredoxin Bcp (Thioredoxin reductase) (Bacterioferritin comigratory protein homolog)	
197716..198792	-	1077	*perM*	R	membrane protein PerM family	
198870..199025	+	156			hypothetical protein	
199053..200615	-	1563	*guaA*	F	GMP synthase [glutamine-hydrolyzing]	
200698..202161	-	1464	*guaB*	F	Inosine-5'-monophosphate dehydrogenase	
202527..203327	+	801	*ugd*	I	Glycerophosphodiester phosphodiesterase	
203643..204071	+	429	*ndk*	F	Nucleoside diphosphate kinase	
204075..205190	+	1116	*rlmN*	J	Dual-specificity RNA methyltransferase RlmN	
205322..206080	+	759	*pilF*	N	Type IV pilus biogenesis protein PilF	
206083..207360	+	1278	*hisS*	J	Histidine--tRNA ligase	
207357..207992	+	636		T	transmembrane protein	
208017..209174	+	1158	*bamB*	M	Outer membrane protein assembly factor BamB	
209239..210636	+	1398	*der*	R	GTPase Der	
210659..211459	-	801	*dam*	L	DNA adenine methylase	
211664..213868	+	2205			ABC transporter ATP-binding component	
213833..214633	-	801	*rsmJ*	J	Ribosomal RNA small subunit methyltransferase J	
214630..215187	-	558		I	Phospholipid N-methyltransferase-like protein	
215347..216399	-	1053	*hisC*	E	Histidinol-phosphate aminotransferase	
216641..217432	+	792	*coaX*	H	Type III pantothenate kinase	
217452..218255	-	804		R	Putative lipoprotein transmembrane	
218395..220275	+	1881	*frgA*	P	Siderophore biosynthesis protein	

220279..221460	+	1182		G	Transporter major facilitator family	
221471..221860	+	390		I	GtrA family protein	
222251..222949	+	699			Uncharacterized protein	
226167..227153	+	987	*intD*	X	Putative phage integrase	
227411..229555	-	2145		S	Uncharacterized protein	
229908..232892	-	2985		R	Uncharacterized protein	
233219..234058	+	840		R	Phenazine biosynthesis protein PhzF family	
234339..234935	-	597			hypothetical protein	
234994..235839	+	846		X	phage integrase	
236033..236158	+	126			hypothetical protein	
236217..236735	-	519			Uncharacterized protein	
236791..238101	-	1311			Uncharacterized protein	
238790..239017	-	228			hypothetical protein	
239282..239488	+	207			hypothetical protein	
240916..252564	-	11649		R	Rhs family protein	
252649..255003	-	2355		R	Uncharacterized protein	
255612..256256	+	645			Putative uncharacterized protein	
256240..257931	+	1692		U	conjugal transfer protein TraD	
258508..259242	+	735			hypothetical protein	
259547..259870	+	324	*virB2*	U	Type IV secretion system protein VirB2	
259895..260194	+	300	*virB3*	U	Plasmid conjugal transfer protein TrbD/VirB3	
260213..262636	+	2424	*virB4*	U	Type IV secretion system protein VirB4	
262630..263331	+	702	*virB5*	U	Type IV secretion system protein VirB5	
263356..263475	-	120			hypothetical protein	
263501..263875	+	375			hypothetical protein	
263872..264882	+	1011	*virB6*	U	Legionella vir homologue protein	
264883..265626	+	744	*virB8*	U	VirB8 family protein	
266348..267433	+	1086	*virB10*	U	Type IV secretion system protein B10	
267440..268411	+	972	*virB11*	U	Type IV secretion system protein VirB11	
268434..269393	-	960		X	transposase	
269562..269966	-	405		T	Putative sensory histidine-kinase / response regulator	
270082..270528	-	447		K	Transcriptional regulator, LuxR family	
271009..273081	-	2073			hypothetical protein	
273099..273965	-	867		L	DNA modification methylase	
273979..274644	-	666			bacteriophage protein	
274638..275690	-	1053			Putative uncharacterized protein	
275694..276611	-	918			Putative uncharacterized protein	
277019..278149	+	1131		X	site-specific recombinase phage integrase family	
278849..282289	+	3441	*znuC*	I	AMP-dependent synthetase and ligase	
282305..283600	-	1296		L	Putative ATPase	
283660..284550	-	891	*rimK*	H	Ribosomal protein S6 modification protein 1	
284560..284988	-	429	*rimK*	S	S6 modification enzyme RimK	
284990..285382	-	393	*sspB*	R	Stringent starvation protein B	
285387..286043	-	657	*sspA*	O	K03599 stringent starvation protein A	
286686..287126	-	441	*rpsI*	J	30S ribosomal protein S9	x
287142..287576	-	435	*rplM*	J	50S ribosomal protein L13	x
287921..288922	+	1002	*hemB*	H	Delta-aminolevulinic acid dehydratase	
289138..290475	+	1338	*tig*	O	Trigger factor	
290561..291226	+	666	*clpP*	O	ATP-dependent Clp protease proteolytic subunit	
291392..292651	+	1260	*clpX*	O	ATP-dependent Clp protease ATP-binding subunit ClpX	
292817..295282	+	2466	*lon*	O	Lon protease	
295409..295699	+	291	*hupB*	L	Nucleoid DNA-binding protein	
295942..297390	+	1449	*ppiD*	O	peptidyl-prolyl cis-trans isomerase	
297385..299613	-	2229		T	Protein kinase	
299654..300349	-	696	*ispD*	I	2-C-methyl-D-erythritol 4-phosphate cytidylyltransferase	
300376..301182	+	807	*cysQ1*	P	3'(2') 5'-bisphosphate nucleotidase	
301318..302331	+	1014	*mdh*	C	Malate dehydrogenase	
302474..304735	-	2262	*ftsK*	D	DNA translocase FtsK	
304927..305883	+	957	*trxB*	O	Thioredoxin reductase	
305915..306649	+	735	*vacJ*	M	Surface lipoprotein	
306695..307174	+	480	*dps*	P	Ferritin Dps family protein	
307423..307824	+	402	*rbp*	J	RNA-binding protein	
307863..308603	-	741	*comF*	R	Competence protein F	
308736..310571	+	1836	*glmS*	M	Glutamine--fructose-6-phosphate aminotransferase [isomerizing]	
310588..311874	-	1287	*glmM*	G	Phosphoglucosamine mutase	
311944..313860	-	1917	*ftsH*	O	ATP-dependent zinc metalloprotease FtsH	
314129..314770	-	642	*rlmE*	J	Ribosomal RNA large subunit methyltransferase E	
314760..315977	-	1218		G	Major facilitator family transporter	
316110..317339	-	1230	*phtH*	G	Putative Major facilitator family transporter	
317833..318333	-	501	*ispF*	I	2-C-methyl-D-erythritol 2 4-cyclodiphosphate synthase	
318538..319116	+	579		K	UPF0301 protein MASE_14395	
319113..319535	+	423	*yneN*	K	Putative Holliday junction resolvase	
319570..321060	+	1491	*mviN*	M	Virulence factor mviN	
321057..322118	+	1062	*ribF*	H	Bifunctional flavokinase/ FAD synthetase	

322325..323065	+	741		M	Putative uncharacterized protein
323317..325344	+	2028		I	Phosphatidylcholine-sterol acyltransferase
325387..326130	+	744	*poxF*	C	Phenol hydroxylase
326166..326654	-	489	*ribH*	H	6 7-dimethyl-8-ribityllumazine synthase
326675..327886	-	1212	*ribBA*	H	Riboflavin biosynthesis protein RibBA
327931..329061	-	1131	*ribD*	H	Riboflavin biosynthesis protein RibD
329124..330473	-	1350	*mpl*	M	UDP-Nacetylmuramate:l-alanyl-gamma-D-glutamyl-m es o-diaminopimelate ligase
330587..331852	+	1266	*pfkA*	G	Phosphofructokinase
331948..332658	+	711	*ymfK*	K	P22 repressor protein c2
332689..333318	-	630	*acpS*	H	4'-phosphopantetheinyl transferase
333507..335171	+	1665	*pilB*	N	Type IV-A pilus assembly ATPase PilB
335186..336388	+	1203	*pilC*	N	Type 4 fimbrial assembly protein
336389..337189	+	801	*pilD*	N	Type 4 prepilin-like proteins leader peptide-processing enzyme
337283..337588	-	306		K	Putative DNA-binding protein
337585..337953	-	369		S	Uncharacterized protein
338284..339276	+	993	*gpsA*	C	Glycerol-3-phosphate dehydrogenase [NAD(P)+]
339335..341269	-	1935	*acnA*	C	Aconitate hydratase
341533..342051	+	519	*dsbB*	O	Disulfide bond formation protein B
342566..343159	-	594	*ygfA*	S	Integral membrane protein
343143..344078	-	936	*mccF*	M	Putative LD-carboxypeptidase
344090..344881	-	792		I	Short-chain dehydrogenase/reductase SDR
344902..347322	-	2421	*gyrB*	L	DNA gyrase subunit B
347309..347461	-	153			hypothetical protein
347658..350273	+	2616	*alaS*	J	Alanine--tRNA ligase
350304..351527	+	1224	*lysC*	E	Aspartokinase
351791..352021	+	231	*csrA*	T	Carbon storage regulator homolog
352565..352807	+	243			hypothetical protein
352949..353854	-	906			Uncharacterized protein
355081..355464	+	384			hypothetical protein
355461..356312	-	852			hypothetical protein
356462..357025	-	564		R	Chitinase class I family protein
357129..357887	+	759		H	Ubiquinone/menaquinone biosynthesis C-methylase UbiE
357935..359080	+	1146	*hemN*	H	Coproporphyrinogen III oxidase
359085..359801	-	717	*trmB*	J	tRNA (guanine-N(7)-)-methyltransferase
359798..360463	-	666	*elbB*	Q	ThiJ/PfpI domain protein
360598..362343	+	1746	*arnT*	M	Undecaprenyl-diphospho-4-amino-4-deoxy-L-arabinose--lipid A 4-amino-4-deoxy-L-arabinose transferase putative
362340..363290	+	951	*arnC*	M	Glycosyl transferase family 2
363371..364357	+	987	*arnD*	G	Polysaccharide deacetylase
364342..365058	-	717	*waaE*	J	Putative gnat family acetyltransferase
365114..366136	+	1023	*arnA*	M	dTDP-glucose 4-6-dehydratase
366096..367208	-	1113	*lpxK*	M	Tetraacyldisaccharide 4'-kinase
367288..368148	-	861	*pdxY*	H	Pyridoxamine kinase
368430..368570	+	141			hypothetical protein
368700..369983	+	1284	*phtJ*	G	Sugar phosphate permease
370183..370587	-	405			hypothetical protein
370608..371534	-	927	*ygfZ*	O	tRNA-modifying protein YgfZ
371707..372234	+	528		S	spore Coat Protein U domain family
372459..372887	+	429		S	Protein U
373114..373614	+	501		S	Spore coat protein U
373648..374361	+	714		N	Putative pili assembly chaperone transmembrane protein
374324..376765	+	2442	*fimD*	U	Putative outer membrane usher transmembrane protein
376756..377262	+	507		S	Putative secreted pili protein
377427..377921	+	495		S	putative spore coat protein
378162..385625	+	7464		U	Probable adhesin
386155..386283	-	129			hypothetical protein
388298..390313	-	2016	*rpoD*	K	RNA polymerase sigma factor RpoD
390418..391749	+	1332	*folKP*	H	2-amino-4-hydroxy-6-hydroxymethyldihydropteridine pyrophosphokinase
393236..394600	-	1365	*gcvPA*	E	Probable glycine dehydrogenase (decarboxylating) subunit 1
394602..394997	-	396	*gcvH*	E	Glycine cleavage system H protein
395030..396121	-	1092	*gcvT*	E	Aminomethyltransferase
396257..397195	-	939		G	Glycosyl transferase family 2
397198..398085	-	888	*waaV*	G	Glycosyl transferase group 2 family protein
398088..398954	-	867	*parB*	D	Chromosome segregation protein Spo0J contains ParB-like nuclease domain
398951..399817	-	867	*parA*	D	Chromosome partition protein
399890..400861	+	972	*ytfM*	P	Periplasmic solute binding protein
400842..401654	+	813	*znuB*	P	ABC Mn2+/Zn2+ transporter ATPase subunit
401611..402429	+	819	*znuA*	P	Putative ABC transporter permease component
402469..403116	-	648		T	Integral membrane sensor signal transduction histidine kinase

403291..404382	-	1092	*nrdB*	F	Ribonucleoside-diphosphate reductase	
404426..407317	-	2892	*nrdA*	F	Ribonucleoside-diphosphate reductase	
408016..409398	+	1383	*algC*	G	Phosphomannomutase	
409549..410319	+	771	*exoA*	L	Exodeoxyribonuclease III	
410240..411646	-	1407			hypothetical protein	
412334..412585	+	252	*infA*	J	Translation initiation factor IF-1	
412569..414884	-	2316	*clpA*	O	ATP-dependent Clp protease ATP-binding subunit ClpA	
415151..416437	-	1287	*icd*	C	Isocitrate dehydrogenase [NADP]	
416627..417073	+	447			hypothetical protein	
417141..417776	+	636		O	Peptidase M50	
417794..418609	+	816	*scpA*	L	Segregation and condensation protein	
418606..419358	+	753	*bacC*	I	Putative oxidoreductase short-chain dehydrogenase/reductase family protein	
419499..421049	+	1551		T	Mitogen-activated protein kinase kinase	
421046..421867	-	822	*xapA*	F	Purine nucleoside phosphorylase	
421874..422227	-	354	*yggT*	O	Probable Fe(2+)-trafficking protein	
422237..423346	-	1110	*mutY*	L	A / G specific adenine glycosylase	
423391..425064	-	1674		M	Uncharacterized protein involved in outer membrane biogenesis AsmA family protein	
425093..425413	-	321		S	structural toxin protein	
425452..426378	+	927		R	Uncharacterized protein	
426524..427192	+	669			Uncharacterized protein	
427510..428085	+	576		F	Ham1 family	
428089..428202	+	114			hypothetical protein	
428192..429505	+	1314		R	General substrate transporter	
429483..430388	-	906	*miaA*	J	tRNA dimethylallyltransferase	
430405..432261	-	1857	*mutL*	L	DNA mismatch repair protein MutL	
432342..432455	-	114			hypothetical protein	
432751..432996	-	246			hypothetical protein	
433087..434130	-	1044	*gtrA*	M	GtrA family protein selenocysteine-containing	
434324..434794	-	471	*yijK*	J	ATPase YjeE family	
434808..436463	-	1656	*yigN*	R	Putative ABC transporter ATP-binding protein	
436700..438037	+	1338	*glyA*	E	Serine hydroxymethyltransferase	
438093..438593	+	501	*nrdR*	K	Transcriptional repressor NrdR	
438571..439011	+	441	*nusB*	K	N utilization substance protein B homolog	
439016..439984	+	969	*thiL*	H	Thiamine-monophosphate kinase	
439991..440509	+	519	*pgpA*	I	Phosphatidylglycerophosphatase	
440867..441127	+	261			hypothetical protein	
441153..441290	+	138			hypothetical protein	
441508..441708	-	201		R	transporter fused subunits of ABC superfamily: ATP-binding components	
441836..442306	-	471	*rlmH*	J	Ribosomal RNA large subunit methyltransferase H	
442306..442701	-	396	*rsfS*	J	Ribosomal silencing factor RsfS	
442759..443430	-	672	*nadD*	H	Probable nicotinate-nucleotide adenylyltransferase	
443515..444537	-	1023	*holA*	L	DNA polymerase III delta subunit	
444544..445074	-	531	*rlpB*	M	Lipoprotein B	
445085..447589	-	2505	*leuS*	J	Leucine--tRNA ligase	
447748..449232	-	1485	*lnt*	M	Apolipoprotein N-acyltransferase	
449241..450233	-	993	*sohB*	O	Non-proteolytic protein peptidase family S49	
451084..451917	+	834	*kdsA*	M	2-dehydro-3-deoxyphosphooctonate aldolase	
451950..453251	+	1302	*eno*	G	Enolase	
453244..453540	+	297	*ftsB*	D	Cell division protein FtsB	
453749..454474	-	726		K	helix-turn-helix domain protein	
455225..455668	+	444			Autoinducer-binding domain protein	
455722..456600	+	879			Uncharacterized protein	
456581..457621	+	1041		T	PAS domain S-box/His Kinase A (Phosphoacceptor) domain/ATPase histidine kinase- DNA gyrase B- and HSP90-like domain/response regulator receiver domain/Hpt domain protein	
457664..458587	+	924		X	Transposase	
458612..459307	-	696	*lolD*	M	Lipoprotein releasing system ATP-binding protein	
459300..460556	-	1257	*lolC*	M	Outer membrane-specific lipoprotein transporter subunit membrane component of ABC superfamily	
460589..461392	-	804	*fabI*	I	Enoyl-[acyl-carrier-protein] reductase [NADH]	
461404..462042	-	639	*udk*	F	Uridine kinase	
462272..463006	-	735			hypothetical protein	
463051..464388	-	1338			hypothetical protein	
464825..466594	+	1770			TonB-dependent siderophore receptor	
466638..467669	-	1032	*ispB*	I	Farnesyltranstransferase	
467791..468192	+	402	*rpsF*	J	30S ribosomal protein S6	x
468277..468462	+	186	*rpsR*	J	30S ribosomal protein S18	x
468482..469366	+	885		S	Hypothetical membrane spanning protein	
469420..469872	+	453	*rplI*	J	50S ribosomal protein L9	x
470017..471390	+	1374	*dnaB*	L	Replicative DNA helicase	
471387..472490	+	1104	*alr*	M	Alanine racemase	

472525..472884	+	360		M	17 kDa surface antigen	
472910..474349	+	1440	*dacB*	M	D-alanyl-D-alanine carboxypeptidase/D-alanyl-D-alanine-endopeptidase	
474395..474538	+	144			hypothetical protein	
474659..475246	-	588	*yfiH*	H	5-formyltetrahydrofolate cyclo-ligase	
475637..476206	+	570		S	YecA family protein	
476240..477538	+	1299	*pepP*	E	Proline aminopeptidase P II	
477535..478818	+	1284	*ubiH*	H	2-polyprenyl-6-methoxyphenol 4-hydroxylase	
478805..480493	+	1689	*sfcA*	C	Malate dehydrogenase (Oxaloacetate-decarboxylating)	
480465..481709	+	1245	*visC*	H	Putative VisC protein	
481698..481820	-	123			hypothetical protein	
481873..482133	-	261			hypothetical protein	
482237..482800	+	564	*gmhB*	E	D D-heptose 1 7-bisphosphate phosphatase	
482793..484187	+	1395	*radA*	O	DNA repair protein radA	
485126..485494	+	369			Sensory box histidine kinase/response regulator	
485625..486578	-	954		I	Esterase/lipase/thioesterase	
486578..487384	-	807		I	Methyltransferase type 11	
487469..487867	+	399	*cdd*	F	Cytidine deaminase	
487911..490001	+	2091		T	Putative sensory histidine-kinase / response regulator	
490060..490506	-	447			Stress responsive A/B barrel domain protein	
490676..490807	-	132			hypothetical protein	
490835..491482	-	648			hypothetical protein	
491664..492395	-	732			Uncharacterized protein	
492907..493062	+	156			hypothetical protein	
495015..495329	-	315	*fis*	K	DNA-binding protein Fis	
495668..497245	+	1578	*dsbD*	O	Thiol:disulfide interchange protein DsbD	
498293..500551	-	2259	*parC*	L	DNA topoisomerase 4 subunit A	
500763..501356	-	594		M	Lipid A deacylase	
501584..501838	+	255	*rpmE*	J	50S ribosomal protein L31	x
501954..503243	+	1290	*phtE*	G	Putative major facilitator family transporter	
503334..504527	-	1194	*aspC*	E	Aminotransferase class I and II	
507218..509173	+	1956	*thrS*	J	Threonine--tRNA ligase	
509191..509769	+	579	*infC*	J	Translation initiation factor IF-3	
509807..510001	+	195	*rpmI*	J	50S ribosomal protein L35	
510033..510389	+	357	*rplT*	J	50S ribosomal protein L20	x
510465..511184	-	720			hypothetical protein	
511296..511904	-	609	*recR*	L	Recombination protein RecR	
511937..512290	-	354	*ybaB*	R	Nucleoid-associated protein Entcl_3319	
512377..513954	-	1578	*dnaX*	L	DNA polymerase III subunits gamma and tau	
514474..516048	+	1575	*prfC*	J	Peptide chain release factor 3	
516094..517308	+	1215		T	Signal transduction histidine kinase	
517305..518027	-	723	*minC*	D	Probable septum site-determining protein MinC	
518211..519872	+	1662	*fadD*	I	AMP-dependent synthetase and ligase	
520094..521449	-	1356	*qseC*	T	Sensory histidine kinase in two-component regulatory system with QseB	
521512..522183	-	672	*qseB*	T	Transcriptional regulatory protein	
522461..524482	-	2022	*ligA*	L	DNA ligase	
524475..525137	-	663	*zipA*	D	Cell division protein ZipA	
525297..528827	-	3531	*smc*	D	Chromosome partition protein Smc	
528981..529274	-	294		S	17 kda surface antigen	
529314..529436	-	123			hypothetical protein	
529435..529890	+	456	*dut*	F	Deoxyuridine 5'-triphosphate nucleotidohydrolase	
529928..530845	-	918			hypothetical protein	
531167..531892	+	726			hypothetical protein	
531873..532856	-	984	*corC*	P	Mg2+/Co2+ transporter	
532837..533259	-	423	*ybeY*	J	Endoribonuclease YbeY	
533276..534247	-	972	*phoH*	T	Phosphate starvation-inducible protein PhoH predicted ATPase	
534244..535572	-	1329	*miaB*	J	(Dimethylallyl)adenosine tRNA methylthiotransferase MiaB	
535608..535760	-	153			hypothetical protein	
535796..537412	-	1617	*gatB*	J	Aspartyl/glutamyl-tRNA(Asn/Gln) amidotransferase subunit B	
537412..538866	-	1455	*gatA*	J	Glutamyl-tRNA(Gln) amidotransferase subunit A	
538883..539170	-	288	*gatC*	J	Aspartyl/glutamyl-tRNA(Asn/Gln) amidotransferase subunit C	
539302..540342	+	1041	*mreB*	D	Rod shape-determining protein MreB	
540400..541224	+	825	*mreC*	D	Cell shape-determining protein MreC	
541355..541840	+	486	*mreD*	M	Rod shape-determining transmembrane protein	
542344..542589	+	246			hypothetical protein	
542549..542797	+	249			hypothetical protein	
542953..544065	+	1113		M	Heptosyltransferase III	
544144..544908	+	765	*waaE*	M	Glycosyl transferase	
544918..546144	+	1227		M	O-antigen ligase	
546302..549310	+	3009		S	Membrane protein-like protein	
549550..551001	+	1452	*tldD*	R	TldD protein	

551083..552816	+	1734	*msbA*	V	Phospholipid-lipopolysaccharide ABC transporter
552877..552999	+	123			hypothetical protein
553022..554011	+	990	*rfe*	M	UDP-GlcNAc:undecaprenyl-phosphate GlcNAc-1-phosphate transferase
554131..554328	-	198			hypothetical protein
554533..555375	+	843		S	UPF0276 protein LLO_0755
555359..556135	+	777		S	Uncharacterized protein
556135..557787	+	1653	*pyrG*	F	CTP synthase
557837..558709	+	873	*rfbD*	G	Lipopolysaccharide transport system permease protein
558722..559471	+	750	*rfbE*	G	ABC transporter related protein
560998..561129	+	132			hypothetical protein
562781..564016	-	1236		H	2-polyprenyl-3-methyl-5-hydroxy-6-metoxy-1_4-benzoquinol methylase
564038..566485	-	2448	*wbkB*	M	Glycosyltransferase
566497..567471	-	975	*gmd*	M	GDP-mannose 4 6-dehydratase
567618..568748	+	1131	*wbpY*	M	Glycosyl transferase group 1
568724..570223	-	1500	*wbpW*	M	WbpW
570471..571601	+	1131		M	Glycosyl transferase group 1
571598..572530	-	933			hypothetical protein
572596..573882	-	1287	*purA*	F	Adenylosuccinate synthetase
573879..574763	-	885	*hflC*	O	Protein HflC
574760..575938	-	1179	*hflK*	O	HflK protein
576239..577408	-	1170	*hflX*	J	GTPase HflX
577401..577664	-	264	*hfq*	T	RNA-binding protein Hfq
577881..581027	-	3147		R	FOG: Ankyrin repeat
581183..583972	-	2790	*polA*	L	DNA polymerase I
584094..585158	+	1065	*guaC*	F	GMP reductase
585230..585604	+	375	*ccmE*	O	Cytochrome c-type biogenesis protein CcmE
585777..586313	+	537			hypothetical protein
586411..586698	+	288		O	Response regulator receiver domain protein
586688..587119	+	432			putative lipoprotein
587178..587570	+	393	*erpA*	O	Putative iron-sulfur cluster insertion protein ErpA
587512..588234	-	723	*yggE*	S	oxidative stress defense protein
588320..589435	-	1116	*anmK*	M	Anhydro-N-acetylmuramic acid kinase
590519..590641	+	123			hypothetical protein
590641..591282	+	642		M	Uncharacterized protein
591496..592503	+	1008		R	WD-40 repeat protein
592839..593294	+	456	*mraZ*	J	cell division protein MraZ
593325..594260	+	936	*rsmH*	J	Ribosomal RNA small subunit methyltransferase H
594257..594604	+	348	*ftsL*	D	Cell division protein FtsL
594620..596368	+	1749	*ftsI*	D	Penicillin-binding protein
596511..597737	+	1227			hypothetical protein
597867..598847	+	981		E	Lysine 2 3-aminomutase
598985..600367	+	1383	*fumC*	C	Fumarate hydratase class II
600377..600724	-	348			hypothetical protein
600769..601143	-	375			hypothetical protein
601118..601333	-	216			hypothetical protein
601396..601587	-	192			hypothetical protein
601930..602082	-	153			hypothetical protein
602039..603064	+	1026		T	Serine/threonine protein kinase
603098..603934	-	837	*dapF*	E	Diaminopimelate epimerase
604052..605317	-	1266	*lysA*	E	Diaminopimelate decarboxylase
605320..605460	-	141			Uncharacterized protein
605558..606232	+	675		R	Carboxylesterase
606281..608074	+	1794	*lepA*	J	Elongation factor 4
608098..608874	+	777	*lepB*	U	Signal peptidase I
609043..609735	+	693	*rnc*	K	Ribonuclease 3
609822..610742	+	921	*era*	J	GTPase Era
611003..611716	+	714			hypothetical protein
611877..612323	-	447		S	Uncharacterized protein
612326..614284	-	1959	*ydaL*	E	Amino acid transporter-like transmembrane protein
615943..616908	+	966	*gshB*	H	Glutathione synthetase
616983..617324	-	342	*sufT*	O	Iron sulfur cluster assembly protein (SufT)
617330..617815	-	486	*nifU*	O	SUF system FeS assembly protein NifU family
617778..620375	-	2598	*sufS*	E	Cysteine desulfurase
620381..621136	-	756	*sufC*	O	FeS assembly ATPase SufC
621304..622746	-	1443	*sufB*	O	FeS assembly protein SufB
622743..623198	-	456	*sufA*	K	Iron sulfur cluster assemblytranscriptional regulator SufA
623598..631805	+	8208	*secA*	U	Preprotein translocase subunit SecA (ATPase- RNA helicase)
631781..635350	+	3570			Threonine--tRNA ligase
635622..636245	-	624			hypothetical protein
636288..637043	-	756		I	Short-chain dehydrogenase/reductase SDR
637069..638253	+	1185		R	Uncharacterized protein
638334..638597	+	264			hypothetical protein

638605..639180	+	576	*icmQ*	U	Component of the Dot/Icm secretion system
639324..640445	+	1122	*icmP*	U	Component of Dot/Icm secretion system. ATPase component
640445..642805	+	2361	*icmO*	U	Component of the Dot/Icm secretion system
642762..642926	-	165			hypothetical protein
642998..643552	+	555	*icmN*	U	Component of the Dot/Icm secretion system
643946..644584	+	639	*icmL*	U	Component of the Dot/Icm secretion system
644658..645578	+	921	*icmK*	U	Component of the Dot/Icm secretion system
645587..648106	+	2520	*icmE*	U	Component of the Dot/Icm secretion system. Major component of a channel
648144..648848	+	705	*icmG*	U	Component of the Dot/Icm secretion system
648854..649468	+	615	*icmC*	U	Component of the Dot/Icm secretion system
649533..649949	+	417	*icmD*	U	Component of the Dot/Icm secretion system
650145..650570	+	426	*icmD*	U	Component of the Dot/Icm secretion system
650581..651177	+	597	*icmJ*	U	Component of the Dot/Icm secretion system
651189..654209	+	3021	*icmB*	U	Component of the Dot/Icm secretion system
654351..654470	-	120			hypothetical protein
654446..656365	+	1920			Putative uncharacterized protein
656439..657191	+	753			NADH:flavin oxidoreductase/NADH oxidase
657198..657470	-	273		T	Morphogene BolA protein
657467..658102	-	636	*ispZ*	D	Probable intracellular septation protein A
658079..658738	+	660	*sua5*	J	Putative translation factor
658852..660228	+	1377	*gltX*	J	Glutamate--tRNA ligase 1
660339..660644	-	306			hypothetical protein
660822..660998	+	177			hypothetical protein
660961..661119	+	159			hypothetical protein
661234..661692	-	459			hypothetical protein
663059..663391	-	333			Uncharacterized protein
663544..664503	+	960			Putative uncharacterized protein
664639..664971	+	333			hypothetical protein
665349..665792	+	444		K	hypothetical protein
665930..666382	+	453		T	PAS domain S-box
666754..667386	+	633		T	Two component transcriptional regulator winged helix family
667515..670094	-	2580		Q	Amino acid adenylation
670118..671023	-	906		R	GCN5-related N-acetyltransferase
671074..671187	+	114			hypothetical protein
673506..673898	+	393			Putative uncharacterized protein
673959..674210	+	252			hypothetical protein
674403..674552	+	150			Uncharacterized protein
674569..676098	-	1530			Uncharacterized protein
676221..678503	-	2283	*rne*	J	RNase E
679403..679726	+	324			hypothetical protein
679918..682503	+	2586	*gyrA*	L	DNA gyrase subunit A
682770..683306	+	537	*pyrE*	F	Orotate phosphoribosyltransferase
683435..683947	+	513	*cvpA*	S	Colicin V production protein
683942..684679	-	738	*lpxH*	M	UDP-2 3-diacylglucosamine hydrolase
684796..684936	-	141			hypothetical protein
684937..686286	+	1350	*gltX*	J	Glutamate--tRNA ligase
686270..687667	+	1398	*cysS*	J	Cysteine--tRNA ligase
687715..688230	-	516			hypothetical protein
688568..689926	+	1359			hypothetical protein
690016..690138	+	123			hypothetical protein
690225..691700	-	1476		R	Multidrug resistance protein B
691847..692668	+	822	*enhA*	M	Enhanced entry protein
692669..692818	+	150			hypothetical protein
692803..693354	+	552		S	alkaline phosphatase DedA family
693473..696847	-	3375	*addA*	L	UvrD/REP helicase
696831..699464	-	2634	*lidB*	L	Uncharacterized protein
699920..700291	+	372			putative TonB-dependent siderophore receptor
700383..702497	-	2115			Secreted effector J SseJ
702860..703504	-	645	*tenA*	H	Thiaminase
703509..704339	-	831	*thiD*	H	Phosphomethylpyrimidine kinase
704323..704988	-	666	*thiE*	H	Thiamine-phosphate synthase
705028..705786	-	759	*thiM*	H	Hydroxyethylthiazole kinase
706101..707030	+	930		X	Transposase
707730..708470	+	741	*rluB*	J	Pseudouridine synthase
708535..709314	-	780			hypothetical protein
709457..710533	+	1077			hypothetical protein
710712..711833	+	1122			hypothetical protein
712259..713098	+	840	*sdhC*	C	Succinate dehydrogenase cytochrome b556 subunit
713050..713448	+	399	*sdhD*	C	Succinate dehydrogenase hydrophobic membrane anchor protein
713452..715218	+	1767	*sdhA*	C	Succinate dehydrogenase flavoprotein subunit

715439..716137	+	699	*sdhB*	C	Succinate dehydrogenase and fumarate reductase iron-sulfur protein	
716380..719178	+	2799	*sucA*	C	2-oxoglutarate dehydrogenase E1 subunit	
719230..720441	+	1212	*sucB*	C	Dihydrolipoamide succinyltransferase E2 subunit	
720558..721568	-	1011		R	Ankyrin repeat	
721725..721934	-	210			hypothetical protein	
722199..723374	+	1176	*sucC*	C	Succinyl-CoA ligase [ADP-forming] subunit beta	
723398..724267	+	870	*sucD*	C	Succinyl-CoA ligase [ADP-forming] subunit alpha	
724264..725238	+	975	*cysK*	E	Cysteine synthase	
725354..727015	-	1662			hypothetical protein	
727184..728764	-	1581		R	Putative transporter fused subunits of ABC superfamily: ATP-binding components	
728937..730019	-	1083	*ychF*	J	Ribosome-binding ATPase YchF	
730031..730639	-	609	*pth*	J	Peptidyl-tRNA hydrolase	
730766..731476	-	711	*rplY*	J	50S ribosomal protein L25	x
731593..731715	+	123			hypothetical protein	
731807..732406	+	600			hypothetical protein	
732771..735887	+	3117			Putative cell wall-associated hydrolase protein	
735925..736041	+	117			hypothetical protein	
736019..736978	-	960	*prs*	F	Ribose-phosphate pyrophosphokinase	
737173..738051	-	879	*ispE*	I	4-diphosphocytidyl-2-C-methyl-D-erythritol kinase	
738060..738788	-	729	*lolB*	M	Outer membrane lipoprotein involved in outer membrane biogenesis	
738885..739754	+	870	*rsmE*	J	Ribosomal RNA small subunit methyltransferase E	
739791..742040	+	2250	*rnr*	K	Ribonuclease R	
742044..742790	+	747	*rlmB*	J	23S rRNA (guanosine-2'-O-)-methyltransferase RlmB	
742926..743654	+	729			Peptidase U62 modulator of DNA gyrase	
743680..744504	+	825			hypothetical protein	
744753..745415	+	663			hypothetical protein	
745538..745723	+	186			hypothetical protein	
745843..746310	+	468	*tadA*	J	Cytosine/adenosine deaminase	
746577..747278	+	702	*sirA*	T	GacA-like protein	
747388..747687	+	300			hypothetical protein	
749687..749800	+	114			hypothetical protein	
749815..750363	+	549	*pgsA*	I	CDP-diacylglycerol--glycerol-3-phosphate 3-phosphatidyltransferase	
751154..751384	+	231	*fdxA*	F	Putative ferredoxin	
751384..752076	+	693	*ydfM*	J	Peptidase M22 glycoprotease	
752190..752381	+	192		S	UPF0434 protein Metal_0981	
752392..753411	+	1020	*pheS*	J	Phenylalanine--tRNA ligase alpha subunit	
753413..755800	+	2388	*pheT*	J	Phenylalanine--tRNA ligase beta subunit	
755929..756228	+	300	*ihfA*	L	Integration host factor subunit alpha	
756380..758122	+	1743		T	Response regulator with CheY-like receiver AAA-type ATPase and DNA-binding domains	
758709..758849	+	141			hypothetical protein	
758862..759233	+	372			RCC1 repeats protein	
759261..759710	+	450	*ugpQ*	T	Universal stress protein	
759959..760183	+	225		S	Uncharacterized protein	
760349..761548	-	1200	*rmuC*	L	DNA recombination protein RmuC	
761578..763425	+	1848	*slt*	M	Lytic transglycosylase	
763574..772318	+	8745	*secA*	U	Preprotein translocase subunit SecA (ATPase- RNA helicase)	
772296..775238	+	2943		R	Cof-like hydrolase	
775413..775742	-	330		S	Transport-associated protein	
775808..775939	-	132			hypothetical protein	
776138..776608	-	471	*ssb*	L	Single-stranded DNA-binding protein	
776782..779661	+	2880	*uvrA*	L	UvrABC system protein A	
779847..780134	-	288		L	Endo/excinuclease amino terminal domain protein	
780196..780873	-	678		M	OmpA-like transmembrane domain protein	
781160..781813	-	654		M	OmpA-like transmembrane domain protein	
781974..782087	-	114			hypothetical protein	
782191..782934	-	744		M	Outer membrane protein	
783064..783717	-	654		M	Outer membrane protein A	
783905..784498	-	594	*ompA*	M	OmpA family protein	
784588..785454	+	867	*ubiA*	H	4-hydroxybenzoate octaprenyltransferase	
785451..785942	+	492	*coaD*	H	Phosphopantetheine adenylyltransferase	
785950..786201	+	252	*fdx1*	C	Ferredoxin	
786433..786567	+	135	*rpmF*	J	50S ribosomal protein L32	x
786575..787609	+	1035	*plsX*	I	Phosphate acyltransferase	
787606..788556	+	951	*fabH*	I	3-oxoacyl-[acyl-carrier-protein] synthase 3	
788712..791507	+	2796		S	Putative low-complexity protein	
791573..793633	+	2061		S	pentapeptide repeat-containing protein	
793764..794693	+	930	*fabD*	I	Malonyl CoA-acyl carrier protein transacylase	
794702..795442	+	741	*fabG*	I	3-ketoacyl-(Acyl-carrier-protein) reductase	
795594..795818	+	225	*acpP*	I	Acyl carrier protein	

795861..797168	+	1308	*fabF*	I	3-oxoacyl-[acyl-carrier-protein] synthase 2	
797187..798293	+	1107		D	Aminodeoxychorismate lyase	
798289..798921	+	633	*tmk*	F	Thymidylate kinase	
799053..800045	+	993	*holB*	L	DNA polymerase III delta prime subunit	
800033..800824	+	792	*tatD*	L	DNase TatD family	
800824..802317	+	1494	*add*	F	Adenosine deaminase	
802311..802862	-	552	*folE*	H	GTP cyclohydrolase 1	
802930..803733	-	804	*metQ*	E	DL-methionine transporter subunit periplasmic-binding component of ABC superfamily	
803763..804407	-	645	*metI*	E	ABC-type methionine transport system permease component	
804391..805437	-	1047	*metN*	E	ABC-type methionine transport system ATPase component	
805770..805976	+	207			hypothetical protein	
806028..806201	-	174			hypothetical protein	
806270..806878	-	609	*gst*	O	Glutathione S-transferase domain	
807503..808147	+	645		K	peptidase family S24	
808122..808385	-	264		K	Transcriptional repressor NrdR	
809090..809470	+	381		T	Response regulator receiver protein	
809490..810218	-	729		K	Transcription regulator LuxR family VCA1078	
810324..810698	+	375		K	Transcriptional repressor NrdR	
810731..811921	-	1191		M	Tetratricopeptide repeat family protein	
811923..812216	-	294		S	Uncharacterized protein	
812288..813805	-	1518	*rpsA*	J	30S ribosomal protein S1	x
813970..814653	-	684	*cmk*	F	Cytidylate kinase	
814644..815426	-	783	*pyrF*	F	Orotidine 5'-phosphate decarboxylase	
815433..815873	-	441		D	Sporulation domain protein	
816017..816133	+	117			hypothetical protein	
816149..818230	+	2082			Aerotolerance-like exported protein	
818195..819520	-	1326	*folC*	H	Bifunctional protein FolC	
819644..820531	-	888	*accD*	I	Acetyl-coenzyme A carboxylase carboxyl transferase subunit beta	
820852..821685	+	834			Putative uncharacterized protein	
821711..822667	-	957	*potD*	E	Putrescine-binding periplasmic protein	
822748..823521	-	774	*potC*	E	Spermidine Putrescine ABC transporter permease component potC	
823499..824362	-	864	*potB*	E	Spermidine/putrescine ABC transporter membrane protein	
824352..825449	-	1098	*potA*	E	Spermidine/putrescine import ATP-binding protein PotA	
825599..826336	-	738			Uncharacterized protein	
826420..827364	-	945			hypothetical protein	
827654..828832	-	1179		R	YD repeat protein	
828987..829772	-	786	*truA*	J	tRNA pseudouridine synthase A	
829888..830826	-	939		R	Tol-pal system protein YbgF	
830875..831423	-	549	*pal*	M	Peptidoglycan-associated lipoprotein (19 kDa surface antigen) (PPL)	
831447..832751	-	1305	*tolB*	U	Protein TolB	
832761..834086	-	1326		G	Major facilitator family transporter	
834263..834598	-	336			hypothetical protein	
834571..837087	-	2517		S	Putative low-complexity protein	
837159..837995	-	837	*tolA*	M	TolA colicin import membrane protein	
837999..838376	-	378	*tolR*	U	Membrane spanning protein in TolA-TolQ-TolR complex	
838444..839139	-	696	*tolQ*	U	TolQ	
839280..840335	-	1056	*ruvB*	L	Holliday junction ATP-dependent DNA helicase RuvB	
840386..840502	-	117			hypothetical protein	
840535..841326	-	792		T	Ankyrin repeat domain protein	
841343..841720	-	378		T	Ankyrin repeat domain protein	
841861..842475	-	615	*ruvA*	L	Holliday junction ATP-dependent DNA helicase RuvA	
842472..843041	-	570	*ruvC*	L	Crossover junction endodeoxyribonuclease RuvC	
843143..843859	-	717		K	Probable transcriptional regulatory protein Nhal_0175	
844143..845240	+	1098			hypothetical protein	
845360..846139	-	780		S	Uncharacterized protein	
846151..847926	-	1776	*aspS*	J	Aspartate--tRNA ligase	
847945..848277	-	333		R	type I antifreeze protein	
848659..849666	-	1008		T	Ankyrin repeat-containing protein	
849673..850179	-	507			hypothetical protein	
850393..852123	+	1731	*proS*	J	Proline--tRNA ligase	
852125..852598	+	474		R	Putative uncharacterized protein	
852687..853352	+	666	*radC*	L	UPF0758 protein Noc_0236	
853401..853682	+	282	*rpmB*	J	50S ribosomal protein L28	x
853702..853854	+	153	*rpmG*	J	50S ribosomal protein L33	x
854005..855342	+	1338		F	Xanthine/uracil/vitamin C permease	
855427..856932	-	1506	*lysS*	J	Lysine--tRNA ligase	
857120..858166	-	1047	*prfB*	J	Peptide chain release factor 2	
858363..859547	-	1185		I	Fatty acid desaturase	
859560..859700	-	141			hypothetical protein	
859708..860280	-	573	*yqgF*	S	outer membrane lipoprotein	

860309..861778	-	1470	*nuoN*	C	NADH-quinone oxidoreductase subunit N	
861775..863328	-	1554	*nuoM*	C	NADH-quinone oxidoreductase chain M	
863350..865290	-	1941	*nuoL*	C	NADH-quinone oxidoreductase chain L	
865297..865602	-	306	*nuoK*	C	NADH-quinone oxidoreductase subunit K	
865599..866198	-	600	*nuoJ*	C	NADH-quinone oxidoreductase chain J	
866383..866880	-	498	*nuoI*	C	NADH-quinone oxidoreductase subunit I	
866877..867905	-	1029	*nuoH*	C	NADH-quinone oxidoreductase subunit H	
867895..870315	-	2421	*nuoG*	C	NADH dehydrogenase I chain G	
870507..871784	-	1278	*nuoF*	C	NADH-quinone oxidoreductase F subunit	
871774..872313	-	540	*nuoE*	C	NADH dehydrogenase subunit E	
872399..873652	-	1254	*nuoD*	C	NADH-quinone oxidoreductase subunit D	
873645..874334	-	690	*nuoC*	C	NADH-quinone oxidoreductase subunit C	
874334..874816	-	483	*nuoB*	C	NADH-quinone oxidoreductase subunit B	
874807..875163	-	357	*nuoA*	C	NADH-quinone oxidoreductase subunit A	
875507..876682	-	1176			Uncharacterized protein	
876870..877598	+	729		P	Uncharacterized protein	
877780..878310	-	531	*ahpD*	P	Alkyl hydroperoxide reductase AhpD	
878398..878877	-	480	*ahpC*	V	Alkyl hydroperoxide reductase peroxiredoxin	
879048..879932	+	885	*oxyR*	K	Transcriptional regulator	
879942..880862	-	921			hypothetical protein	
880977..882101	-	1125	*ddl*	M	D-alanine--D-alanine ligase	
883113..883676	+	564		S	Uncharacterized protein	
890320..891423	+	1104			hypothetical protein	
891398..891514	+	117			hypothetical protein	
891481..892065	-	585	*rnhB*	L	Ribonuclease HII	
892149..893342	-	1194	*lpxB*	M	Lipid-A-disaccharide synthase	
893412..894185	-	774	*lpxA*	M	Acyl-(Acyl carrier protein)-UDP-N-acetylglucosamine acyltransferase	
894188..894637	-	450	*fabZ*	I	3-hydroxyacyl-[acyl-carrier-protein] dehydratase FabZ	
894849..895889	-	1041	*lpxD*	M	UDP-3-O-acylglucosamine N-acyltransferase	
896063..896524	-	462	*ompH*	M	Chaperone protein skp	
896714..899056	-	2343	*bamA*	M	Outer membrane protein assembly factor BamA	
899145..900401	-	1257	*dxr*	I	1-deoxy-D-xylulose 5-phosphate reductoisomerase	
900398..901210	-	813	*cdsA*	I	Phosphatidate cytidylyltransferase	
901203..901958	-	756	*uppS*	I	Ditrans polycis-undecaprenyl-diphosphate synthase ((2E 6E)-farnesyl-diphosphate specific)	
901969..902529	-	561	*frr*	J	Ribosome-recycling factor	
902536..903264	-	729	*pyrH*	F	Uridylate kinase	
903505..904392	-	888	*tsf*	J	Elongation factor Ts	
904397..905185	-	789	*rpsB*	J	30S ribosomal protein S2	x
905345..905500	-	156			hypothetical protein	
905736..906551	+	816		Q	Putative taurine catabolism dioxygenase TauD	
906567..907802	+	1236		E	Uncharacterized protein	
907982..908350	+	369			Uncharacterized protein	
908356..909696	+	1341			Uncharacterized protein	
909761..910414	-	654		S	Uncharacterized protein	
910529..911134	+	606	*wrbA*	C	Trp repressor binding protein	
911239..912144	-	906		X	Putative transposase YhgA family protein	
912499..913197	-	699	*hda*	L	DnaA family protein	
913201..913791	-	591	*pgsA2*	I	CDP-alcohol phosphatidyltransferase	
913834..914130	+	297	*ihfB*	L	Integration host factor subunit beta	
914147..915166	-	1020	*obgE*	D	GTPase Obg	
915209..915499	-	291	*rpmA*	J	50S ribosomal protein L27	x
915512..915820	-	309	*rplU*	J	50S ribosomal protein L21	x
916372..917331	+	960	*yhgA*	X	Transposase	
917398..917547	+	150			hypothetical protein	
917615..917776	+	162			hypothetical protein	
917865..918116	-	252			hypothetical protein	
918397..919278	-	882		V	Aminoglycoside/hydroxyurea antibiotic resistance kinase	
919314..920084	-	771		H	4'-phosphopantetheinyl transferase	
920102..922177	-	2076		T	Putative sensory histidine-kinase / response regulator	
922174..925194	-	3021		Q	Amino acid adenylation domain protein	
925402..925830	-	429		T	Putative sensory histidine-kinase / response regulator	
926130..927041	+	912		X	Uncharacterized protein	
927103..927726	-	624	*phzF*	R	Phenazine biosynthesis PhzC/PhzF protein	
927819..928268	+	450		T	Putative sensory box histidine kinase/response regulator	
928328..928642	-	315		R	YciI like protein	
928750..929304	-	555		O	Heat shock protein Hsp20	
929717..930370	-	654			Uncharacterized protein	
930369..930545	+	177		O	SUF system FeS assembly protein NifU family	
930632..931543	-	912			Peptidyl-prolyl cis-trans isomerase	
931595..932044	-	450			hypothetical protein	
932128..932709	-	582			Peptidase	
933067..934980	-	1914			hypothetical protein	
935461..936060	+	600			Uncharacterized protein	

936397..937368	+	972			hypothetical protein
937584..942224	-	4641			Putative uncharacterized protein
942203..944722	-	2520			Uncharacterized protein
944983..945105	-	123			hypothetical protein
945295..945843	-	549	*yrbF*	R	Bacterial transferase hexapeptide repeat protein
945864..946151	-	288			hypothetical protein
946245..946583	-	339			hypothetical protein
946883..948238	+	1356	*rhlE-2*	L	DEAD/DEAH box helicase domain protein
948625..949443	+	819		S	UPF0761 membrane protein rbn
949507..950373	+	867	*pdxS*	H	Pyridoxal biosynthesis lyase PdxS
950376..950951	+	576	*pdxT*	H	Glutamine amidotransferase subunit PdxT
950925..952514	-	1590			hypothetical protein
952659..953897	+	1239	*wecD*	L	Integrase family protein
954137..954325	+	189			hypothetical protein
954334..955248	-	915	*hemF*	H	Coproporphyrinogen-III oxidase aerobic
955301..955723	+	423		S	Uncharacterized protein
955763..955915	-	153	*rubA*	C	Rubredoxin
956078..957067	-	990	*ftsY*	U	Signal recognition particle receptor FtsY
957293..957865	+	573	*rsmD*	J	N6-adenine-specific methylase
957936..958424	+	489	*dotD*	U	Component of the Dot/Icm secretion system. Lipoprotein
958459..959259	+	801	*dotC*	U	Component of the Dot/Icm secretion system.
959234..960364	+	1131	*dotB*	U	Component of the Dot/Icm secretion system.
960357..961361	+	1005	*epmA*	J	Lysyl-tRNA synthetase
962215..963507	+	1293			hypothetical protein
963526..965013	-	1488	*pncB*	H	Nicotinate phosphoribosyltransferase
965197..965688	-	492	*dksA*	J	RNA polymerase-binding transcription factor DksA
965843..966214	+	372			hypothetical protein
966228..967070	-	843	*prmC*	J	Release factor glutamine methyltransferase
967078..967407	-	330			Uncharacterized protein
967575..968657	-	1083	*prfA*	J	Peptide chain release factor 1
968882..969796	+	915			hypothetical protein
969997..970323	+	327			hypothetical protein
970510..971097	-	588			hypothetical protein
972525..973316	+	792	*yraP*	M	ABC transporter related protein
973306..974106	+	801		M	ABC transport system periplasmic substrate binding protein
974173..974781	+	609		R	ABC transporter
974832..975923	+	1092			hypothetical protein
975892..976515	+	624			Putative membrane protein
976801..977301	+	501			Putative membrane protein
977380..978630	-	1251	*hemA*	H	Glutamyl-tRNA reductase
978754..979656	-	903	*prmA*	J	Ribosomal protein L11 methyltransferase
979661..981001	-	1341	*accC*	I	Acetyl-CoA carboxylase biotin carboxylase
981168..981596	-	429	*accB*	I	Biotin carboxyl carrier protein of acetyl-coa carboxylase (Bccp)
981683..983803	+	2121	*recG*	L	ATP-dependent DNA helicase RecG
983782..984621	-	840			Uncharacterized protein
984939..986375	-	1437	*lpdA*	C	Dihydrolipoyl dehydrogenase
986576..987871	-	1296	*aceF*	C	Dihydrolipoamide acetyltransferase component of pyruvate dehydrogenase complex
987913..990588	-	2676	*aceE*	C	Pyruvate dehydrogenase E1 component
990557..990685	-	129			hypothetical protein
990803..991546	-	744	*ampE*	V	transmembrane protein
991592..992764	-	1173	*kefC*	P	Sodium/hydrogen antiporter
992768..995905	-	3138	*putA*	E	Putative bifunctional PutA protein (Proline dehydrogenase/ delta-1-pyrroline-5-carboxylate dehydrogenase)
996073..996411	-	339	*icmS*	U	component of Dot/Icm secretion system. Substrate recognition; presentation to translocon
996421..996681	-	261	*icmT*	U	IcmT
996817..999096	-	2280			hypothetical protein
999284..999451	+	168			Ankyrin repeat-containing protein
999622..999987	-	366		X	Terminase small subunit
1001294..1003654	-	2361		T	Putative sensory histidine-kinase / response regulator
1011534..1012865	+	1332			Type I restriction-modification system M subunit
1012890..1015316	-	2427		T	Putative sensory histidine-kinase / response regulator
1015386..1016948	-	1563		C	Glycerol-3-phosphate dehydrogenase
1016983..1017615	-	633	*hflD*	X	High frequency lysogenization protein HflD homolog
1017650..1018732	-	1083	*mraY*	M	Phospho-N-acetylmuramoyl-pentapeptide-transferase
1018760..1020094	-	1335	*murF*	M	UDP-N-acetylmuramoyl-tripeptide--D-alanyl-D-alanine ligase
1020180..1021304	+	1125		M	Putative glycosyl transferase
1021808..1024756	+	2949		R	ankyrin 2 neuronal isoform 4
1024753..1026237	-	1485	*murE*	M	UDP-N-acetylmuramoyl-L-alanyl-D-glutamate--2 6-diaminopimelate ligase
1026485..1026733	+	249		P	FeoA family protein

1026746..1029085	+	2340	*feoB*	P	Ferrous iron transport protein B
1029078..1029317	+	240			Putative uncharacterized protein
1029447..1029788	+	342	*dgkA*	I	Diacylglycerol kinase DagK
1029779..1031338	+	1560		M	Putative sulfatase
1032933..1033400	+	468			hypothetical protein
1033652..1040023	+	6372		R	AAA ATPase containing von Willebrand factor type A (vWA) domain
1040034..1041047	-	1014	*yjbN*	J	tRNA-dihydrouridine synthase
1041165..1041416	+	252			hypothetical protein
1041598..1043328	-	1731	*recJ*	L	Single-stranded-DNA-specific exonuclease RecJ
1043529..1043900	+	372		S	hypothetical membrane spanning protein
1043963..1044253	-	291		K	Uncharacterized protein
1044246..1044542	-	297		S	Uncharacterized protein
1044614..1045789	-	1176		M	peptidoglycan-binding domain 1 protein
1045798..1047093	-	1296	*ispG*	I	4-hydroxy-3-methylbut-2-en-1-yl diphosphate synthase
1047444..1048331	+	888	*rpoH*	K	RNA polymerase sigma factor RpoH
1048328..1048504	+	177			Putative uncharacterized protein
1048521..1049165	+	645		P	Adenylate cyclase
1049217..1049363	+	147			hypothetical protein
1049472..1049777	+	306		J	Putative uncharacterized protein
1049759..1050415	-	657		R	Endonuclease III
1050479..1051858	-	1380			hypothetical protein
1051970..1053265	-	1296		S	Uncharacterized protein
1053427..1053795	-	369			hypothetical protein
1054523..1055518	-	996	*hemH*	H	Ferrochelatase
1055515..1056315	-	801		V	Antibiotic transport system permease protein
1056430..1057095	+	666		R	Alpha/beta hydrolase
1057092..1058162	+	1071	*yhbH*	R	AFG1-family ATPase
1058189..1058575	-	387	*bph2_1*	L	DNA-binding protein BpH2
1058703..1059512	-	810		S	Putative uncharacterized protein
1059558..1060517	-	960	*erfK*	M	Lipoprotein-anchoring transpeptidase ErfK/SrfK
1060761..1061882	+	1122	*rodA*	D	Rod shape determining protein RodA
1061958..1062863	+	906	*mltB*	M	Membrane bound lytic murein transglycosylase
1062847..1063710	+	864	*rlpA*	M	Rare lipoprotein A
1063765..1065240	+	1476		U	Putative integral membrane protein
1065416..1066228	+	813	*lgt*	M	Prolipoprotein diacylglyceryl transferase
1066320..1067687	+	1368		V	Putative efflux protein MATE family
1067712..1069271	+	1560	*gpmI*	G	2 3-bisphosphoglycerate-independent phosphoglycerate mutase
1069338..1070105	+	768			hypothetical protein
1070364..1071716	+	1353	*ctpA*	O	Carboxy-terminal protease
1071790..1072626	-	837			hypothetical protein
1072764..1073357	-	594			hypothetical protein
1073918..1077763	-	3846		R	FOG: Ankyrin repeat
1078546..1090194	-	11649		R	Rhs family protein
1090279..1092633	-	2355		R	Uncharacterized protein
1093242..1093886	+	645			Putative uncharacterized protein
1093870..1095561	+	1692		U	conjugal transfer protein TraD
1096138..1096872	+	735			hypothetical protein
1097177..1097500	+	324	*virB2*	U	Vbh2
1097525..1097824	+	300	*virB3*	U	Plasmid conjugal transfer protein TrbD/VirB3
1097875..1100265	+	2391	*virB4*	U	Type IV secretion system protein VirB4
1100259..1100960	+	702	*virB5*	U	Legionella vir homologue protein B5
1100985..1101104	-	120			hypothetical protein
1101130..1101504	+	375			hypothetical protein
1101501..1102511	+	1011	*virB6*	U	Type IV secretion system protein B6
1102512..1103255	+	744	*virB8*	U	Type IV secretion system protein B8
1103977..1105062	+	1086	*virB10*	U	Type IV secretion system protein B10
1105069..1106040	+	972	*virB11*	U	Type IV secretion system protein VirB11
1106063..1107022	-	960		X	Transposase
1107191..1107595	-	405		T	Putative sensory histidine-kinase / response regulator
1107711..1108157	-	447		K	Transcriptional regulator, LuxR family
1108638..1110710	-	2073			hypothetical protein
1110728..1111594	-	867		L	DNA modification methylase
1111608..1112273	-	666			bacteriophage protein
1112267..1113319	-	1053			Putative uncharacterized protein
1113323..1114240	-	918			Putative uncharacterized protein
1114648..1115778	+	1131		X	site-specific recombinase phage integrase family
1115968..1117941	-	1974		S	Oligopeptide transporter
1118077..1119348	-	1272	*serS*	J	Serine--tRNA ligase
1119409..1120293	+	885	*folD*	H	Bifunctional protein FolD
1120520..1120825	-	306			Low complexity protein contains internal repeats QQVlQQDVAQLQAG
1121174..1122574	-	1401	*pepB*	E	Leucyl aminopeptidase PepB putative

1123901..1124404	-	504		T	Putative ankyrin repeat protein RF_0381	
1124388..1124717	+	330			hypothetical protein	
1124781..1126541	-	1761	*dnaG*	L	DNA primase	
1126677..1127126	-	450	*lporfX*	S	GatB/Yqey domain protein	
1127139..1127372	-	234	*rpsU*	J	30S ribosomal protein S21	x
1127435..1128487	+	1053	*tsaD*	J	tRNA N6-adenosine threonylcarbamoyltransferase	
1128484..1130475	-	1992			Uncharacterized protein	
1130629..1131225	-	597	*plsY*	I	Glycerol-3-phosphate acyltransferase	
1131328..1131831	+	504	*orn*	A	Oligoribonuclease	
1131921..1132556	+	636	*efp*	J	Elongation factor P	
1132553..1132741	+	189			hypothetical protein	
1132848..1133018	-	171			hypothetical protein	
1133355..1134272	-	918	*truB*	J	tRNA pseudouridine synthase B	
1134308..1134736	-	429	*rbfA*	J	Ribosome-binding factor A	
1134756..1137197	-	2442	*infB*	J	Translation initiation factor IF-2	
1137265..1138776	-	1512	*nusA*	K	Transcription termination/antitermination protein NusA	
1138790..1139221	-	432	*rimP*	J	Ribosome maturation factor RimP	
1139666..1141066	-	1401	*arp*	T	Ankyrin repeat domain protein	
1141078..1141572	-	495			hypothetical protein	
1141623..1142654	-	1032	*asd*	E	Aspartate-semialdehyde dehydrogenase	
1142638..1143585	-	948	*prmB*	J	50S ribosomal protein L3 glutamine methyltransferase	
1143766..1144629	-	864			hypothetical protein	
1144781..1145167	+	387		T	Hpt domain protein	
1145289..1145654	-	309	*rplQ*	J	50S ribosomal protein L17	x
1145669..1146703	-	1035	*rpoA*	K	DNA-directed RNA polymerase subunit alpha	
1146716..1147336	-	621	*rpsD*	J	30S ribosomal protein S4	x
1147367..1147813	-	447	*rpsK*	J	30S ribosomal protein S11	x
1147864..1148223	-	360	*rpsM*	J	30S ribosomal protein S13	x
1148429..1148545	-	117	*rpmJ*	J	50S ribosomal protein L36	x
1148700..1150052	-	1353	*secY*	U	Protein translocase subunit SecY	
1150053..1150487	-	435	*rplO*	J	50S ribosomal protein L15	x
1150477..1150674	-	198	*rpmD*	J	50S ribosomal protein L30	x
1150689..1151192	-	504	*rpsE*	J	30S ribosomal protein S5	x
1151212..1151574	-	363	*rplR*	J	50S ribosomal protein L18	x
1151593..1152072	-	480	*rplF*	J	50S ribosomal protein L6	x
1152138..1152527	-	390	*rpsH*	J	30S ribosomal protein S8	x
1152546..1152851	-	306	*rpsN*	J	30S ribosomal protein S14	x
1152857..1153399	-	432	*rplE*	J	50S ribosomal protein L5	x
1153414..1153734	-	321	*rplX*	J	50S ribosomal protein L24	x
1153748..1154107	-	360	*rplN*	J	50S ribosomal protein L14	x
1154113..1154352	-	240	*rpsQ*	J	30S ribosomal protein S17	x
1154406..1154603	-	198	*rpmC*	J	50S ribosomal protein L29	x
1154612..1155025	-	414	*rplP*	J	50S ribosomal protein L16	x
1155061..1155831	-	771	*rpsC*	J	30S ribosomal protein S3	x
1155834..1156178	-	345	*rplV*	J	50S ribosomal protein L22	x
1156214..1156507	-	294	*rpsS*	J	30S ribosomal protein S19	x
1156544..1157371	-	828	*rplB*	J	50S ribosomal protein L2	x
1157387..1157704	-	318	*rplW*	J	50S ribosomal protein L23	x
1157701..1158318	-	618	*rplD*	J	50S ribosomal protein L4	x
1158353..1159018	-	666	*rplC*	J	50S ribosomal protein L3	x
1159123..1159455	-	333	*rpsJ*	J	30S ibosomal protein S10	x
1159476..1160678	-	1203	*tuf*	J	Elongation factor Tu	
1160707..1162818	-	2112	*fusA*	J	Elongation factor G 2	
1162847..1163392	-	546	*rpsG*	J	30S ribosomal protein S7	x
1163418..1163795	-	378	*rpsL*	J	30S ribosomal protein S12	x
1164066..1166285	-	2220			Predicted oxidoreductase	
1166509..1170894	-	4386	*rpoC*	K	DNA-directed RNA polymerase subunit beta'	
1170942..1175072	-	4131	*rpoB*	K	DNA-directed RNA polymerase subunit beta	
1175254..1175643	-	390	*rplL*	J	50S ribosomal protein L7/L12	x
1175689..1176219	-	531	*rplJ*	J	50S ribosomal protein L10	x
1176261..1176425	-	165			hypothetical protein	
1176608..1177312	-	705	*rplA*	J	50S ribosomal protein L1	x
1177314..1177748	-	435	*rplK*	J	50S ribosomal protein L11	x
1177982..1178602	-	621	*nusG*	K	Transcription termination/antitermination protein NusG	
1178622..1178993	-	372	*secE*	U	Protein translocase subunit SecE	
1179582..1180175	+	594	*yhgN*	E	MarC family integral membrane protein	
1180283..1181530	-	1248	*rocD*	E	Acetylornithine aminotransferase	
1181537..1182406	-	870	*rocF*	E	Arginase	
1182654..1184831	+	2178	*kef*	P	cation:proton antiporter	
1185042..1186370	+	1329	*pcnB*	J	Poly(A) polymerase I	
1186367..1187971	+	1605	*pgi*	G	Glucose-6-phosphate isomerase	
1188136..1189011	+	876		R	decarboxylase family protein	
1189012..1190718	-	1707		T	Ankyrin repeat protein	
1190740..1196613	-	5874			Chromosome segregation ATPase-like protein	
1196645..1197760	-	1116			hypothetical protein	

1198111..1199220	+	1110			hypothetical protein
1199258..1199857	-	600			hypothetical protein
1199888..1202296	-	2409	*dotA*	U	DotA
1202293..1202748	-	456	*icmV*	U	Intracellular multiplication protein IcmV
1203105..1203557	+	453	*icmW*	U	Component of the Dot/Icm secretion system
1203557..1204081	+	525			hypothetical protein
1204103..1205119	+	1017	*icmX*	U	IcmX
1205140..1207020	+	1881	*dxs*	H	1-deoxy-D-xylulose-5-phosphate synthase
1207065..1207211	+	147			hypothetical protein
1207271..1209976	+	2706	*secA*	U	Protein translocase subunit SecA
1209978..1210760	+	783	*map*	J	Methionine aminopeptidase
1211118..1212044	+	927		V	Related to multidrug-efflux transport protein A
1212050..1215148	+	3099		V	Hydrophobic/amphiphilic exporter-1
1215354..1216175	+	822	*dapD*	E	2 3 4 5-tetrahydropyridine-2 6-dicarboxylate N-succinyltransferase
1216232..1217689	+	1458		M	Undecaprenyl-phosphomannose:protein mannosyltransferase
1217757..1219220	+	1464		M	Undecaprenyl-phosphomannose:protein mannosyltransferase
1219497..1219862	-	366	*secG*	U	Putative preprotein translocase SecG Subunit
1219898..1220662	-	765	*tpiA*	G	Triosephosphate isomerase
1220803..1221903	-	1101			Peptidase M23B family
1221908..1222021	+	114			hypothetical protein
1222044..1222934	-	891	*ppnK*	F	Probable inorganic polyphosphate/ATP-NAD kinase
1223169..1223798	+	630	*grpE*	O	heat shock protein GrpE
1223923..1225884	+	1962	*dnaK*	O	Chaperone protein DnaK
1226126..1227259	+	1134	*dnaJ*	O	Chaperone protein DnaJ
1227364..1227825	+	462	*greA*	K	Transcription elongation factor GreA
1227822..1228718	-	897		R	Small GTP-binding protein
1229022..1229837	+	816		M	Putative uncharacterized protein
1229892..1230413	-	522			hypothetical protein
1230483..1231277	-	795	*thyA*	F	Thymidylate synthase
1231905..1232060	+	156			hypothetical protein
1232346..1233683	+	1338	*udg*	M	Nucleotide sugar dehydrogenase
1233680..1234546	+	867	*galU*	M	UTP--glucose-1-phosphate uridylyltransferase
1235929..1236069	+	141			hypothetical protein
1236123..1236845	+	723		H	Generic methyl-transferase
1236829..1237275	+	447	*rnhA*	L	Ribonuclease H
1237454..1238461	-	1008	*lpxC*	M	UDP-3-O-[3-hydroxymyristoyl] N-acetylglucosamine deacetylase
1238464..1239609	-	1146	*ftsZ*	D	Cell division protein FtsZ
1239789..1241036	-	1248	*ftsA*	D	Cell division protein ftsA
1241095..1241880	-	786	*ftsQ*	D	Cell division protein FtsQ
1241990..1245565	-	3576	*dnaE*	L	DNA polymerase III alpha subunit
1245746..1250305	+	4560		R	Uncharacterized protein
1250347..1251171	+	825	*mutM*	L	Formamidopyrimidine-DNA glycosylase
1251188..1256053	-	4866		E	glutamate dehydrogenase
1256232..1256930	+	699	*ubiG*	H	Ubiquinone biosynthesis O-methyltransferase
1256932..1257630	+	699	*gph*	C	Phosphoglycolate phosphatase-like protein
1257685..1258077	+	393	*xerD*	I	Thioesterase superfamily protein
1258074..1258376	-	303	*minE*	D	Cell division topological specificity factor
1258373..1259197	-	825	*minD*	D	Site-determining protein
1259560..1260282	+	723	*recO*	L	DNA repair protein RecO
1260419..1260904	+	486		M	17 kDa surface antigen
1260897..1261016	-	120			hypothetical protein
1261088..1262332	+	1245	*ampG*	R	Na+/melibiose symporter or related transporter
1262329..1262715	-	387		V	Endoribonuclease L-PSP
1263045..1263365	+	321	*groS*	O	10 kDa chaperonin
1263402..1265048	+	1647	*groL*	O	60 kDa chaperonin
1265307..1265999	+	693			hypothetical protein
1266127..1267041	+	915	*htpX*	O	heat shock protein HtpX
1267049..1267894	-	846	*pla*	M	Protease VII (Omptin)
1268077..1270044	-	1968	*yfcX*	I	YfcX enoyl CoA hydratase
1270044..1271327	-	1284		I	Acetyl-CoA acetyltransferase
1271424..1273637	-	2214	*fadE*	I	Acyl-CoA dehydrogenase
1273713..1274189	-	477	*pilX*	N	Uncharacterized protein
1274957..1275298	-	342			hypothetical protein
1275441..1276289	-	849	*pilW*	N	prepilin-type N- cleavage/methylation domain protein
1276286..1276666	-	381	*pilV*	N	Methylation
1276827..1277198	+	372			Uncharacterized protein
1277265..1278755	-	1491	*pepA*	E	Probable cytosol aminopeptidase
1278903..1280015	+	1113	*lptF*	M	Lipopolysaccharide export system permease protein
1280156..1281223	+	1068	*lptG*	M	Lipopolysaccharide export system permease protein
1281378..1282988	+	1611	*nadE*	H	NAD synthase
1284787..1285002	-	216			hypothetical protein

1285137..1286105	+	969	*rluD*	J	Pseudouridine synthase	
1286098..1286829	+	732	*yfhQ*	P	Uncharacterized protein	
1286984..1287541	+	558			Putative uncharacterized protein	
1287739..1288029	-	291			hypothetical protein	
1288244..1289518	-	1275	*corB*	P	Magnesium and cobalt efflux protein	
1290097..1291539	+	1443	*ffh*	U	Signal recognition particle protein	
1291639..1291926	+	288	*rpsP*	J	30S ribosomal protein S16	x
1291993..1292478	+	486	*rimM*	J	Ribosome maturation factor RimM	
1292478..1293209	+	732	*trmD*	J	tRNA (guanine-N(1)-)-methyltransferase	
1293226..1293636	+	411	*rplS*	J	50S ribosomal protein L19	x
1293723..1294163	+	441		L	Methylated-DNA/protein-cysteine methyltransferase	
1294654..1296969	-	2316		R	DNA internalization-related competence protein ComEC/Rec2	
1297170..1298180	-	1011	*pit*	P	Low affinity inorganic phosphate transporter	
1298202..1298321	-	120			hypothetical protein	
1298417..1299550	+	1134	*dapE*	E	Succinyl-diaminopimelate desuccinylase	
1299766..1300482	-	717		M	Uncharacterized protein	
1300548..1301129	-	582		T	Uncharacterized protein	
1301254..1301637	-	384			hypothetical protein	
1301914..1303278	-	1365	*gltA*	C	Citrate synthase	
1303441..1304385	+	945	*rluC*	J	Pseudouridine synthase	
1304382..1305047	-	666	*adk*	F	Adenylate kinase	
1305310..1306089	+	780		L	Predicted 3'-5' exonuclease related to the exonuclease domain of PolB	
1307475..1308911	+	1437	*dacB*	M	D-alanyl-D-alanine carboxypeptidase/D-alanyl-D-alanine-endopeptidase	
1308948..1311158	+	2211	*relA*	T	(P)ppGpp synthetase I SpoT/RelA	
1311216..1312574	+	1359	*xseA*	L	Exodeoxyribonuclease 7 large subunit	
1312697..1315000	-	2304	*topA*	L	DNA topoisomerase 1	
1315003..1316241	-	1239	*smf*	L	SMF protein	
1316309..1316836	-	528	*ppa*	C	Inorganic pyrophosphatase	
1316925..1317266	+	342		R	Histidine triad (HIT) protein	
1317274..1317393	-	120			hypothetical protein	
1318052..1318492	+	441	*holC*	L	DNA polymerase III chi subunit HolC	
1318486..1321260	+	2775	*valS*	J	Valine--tRNA ligase	
1321478..1321912	+	435			Putative exported protein	
1322089..1322808	+	720	*dnaQ*	L	DNA polymerase III epsilon subunit	
1322809..1323975	+	1167	*nhaA*	P	Na(+)/H(+) antiporter NhaA	
1324230..1325300	-	1071	*hemE*	H	Uroporphyrinogen decarboxylase	
1325304..1326440	-	1137	*yeaZ*	S	Hypothetical membrane spanning protein	
1327927..1328445	-	519	*pilP*	N	Type IV pilus biogenesis protein PilP	
1328473..1329321	-	849	*pilM*	N	Type IV fimbrial biogenesis protein involved in pilus assembly	
1329429..1329557	-	129			hypothetical protein	
1329754..1332195	+	2442	*ponA*	M	Fused penicillin-binding protein 1a: murein transglycosylase murein transpeptidase	
1332228..1332659	-	432	*yjgP*	O	Putative thiol-disulfide oxidoreductase	
1333767..1334534	+	768		P	ABC transporter permease	
1334540..1335481	+	942	*thiY*	P	ABC transporter periplasmic substrate-binding component	
1335874..1338051	+	2178	*uvrD*	L	DNA-dependent helicase II	
1338111..1338734	-	624	*yciA*	L	Smr protein/MutS2-like protein	
1338851..1340020	+	1170		M	LppC lipofamily protein	
1340020..1340424	+	405		L	UPF0102 protein Nwat_2748	
1340428..1341015	+	588	*gmhA*	G	Phosphoheptose isomerase	
1341017..1341706	+	690		M	D-alanyl-D-alanine dipeptidase	
1341843..1341992	-	150			hypothetical protein	
1341980..1342969	+	990			Major facilitator superfamily Na-driven efflux pump	
1342989..1343852	-	864			hypothetical protein	
1343911..1344561	-	651		G	Phosphoglycerate mutase	
1344573..1345361	-	789	*kdsB*	M	3-deoxy-D-manno-octulosonate cytidylyltransferase	
1347535..1347702	+	168		S	UPF0391 membrane protein Cseg_4044	
1347736..1348902	-	1167	*tgt*	J	Queuine tRNA-ribosyltransferase	
1348895..1349647	-	753	*yeeA*	J	RNA methyltransferase TrmH family group 1	
1349747..1350550	+	804	*suhB*	G	inositol monophosphatase	
1350567..1351478	-	912	*secF*	U	Protein-export membrane protein SecF	
1351491..1353353	-	1863	*secD*	U	Protein translocase subunit SecD	
1353518..1353853	-	336	*yajC*	U	Protein translocase subunit yajC	
1353979..1354995	-	1017	*queA*	J	S-adenosylmethionine:tRNA ribosyltransferase-isomerase	
1361522..1362544	+	1023	*nagZ*	G	Beta-hexosaminidase	
1362554..1363120	+	567	*ppt*	F	Hypoxanthine-guanine phosphoribosyltransferase	
1363170..1363502	+	333	*trxA*	O	Thioredoxin	
1363770..1365035	+	1266	*rho*	K	Transcription termination factor Rho	
1365097..1365687	-	591		O	Putative inner membrane protein	
1365692..1365844	-	153			hypothetical protein	
1365922..1366866	-	945	*sua5*	J	Sua5/YciO/YrdC/YwlC family protein	

1367059..1367784	+	726		O	Uncharacterized protein
1367799..1368881	+	1083	*mnmA*	J	tRNA-specific 2-thiouridylase MnmA
1368868..1370238	+	1371	*murD*	M	UDP-N-acetylmuramoylalanine--D-glutamate ligase
1370240..1371388	+	1149	*ftsW*	D	Lipid II flippase FtsW
1371471..1372889	+	1419	*murC*	M	UDP-N-acetylmuramate--L-alanine ligase
1372893..1373774	+	882	*murB*	M	UDP-N-acetylenolpyruvoylglucosamine reductase
1373769..1375334	-	1566		E	Putative amino acid transporters
1375421..1376143	-	723	*rph*	J	Ribonuclease PH
1376230..1377081	+	852	*yhcM*	S	YicC-like domain-containing protein
1377085..1377717	+	633	*gmk*	F	Guanylate kinase
1377979..1378380	+	402		I	Putative nuclease
1378353..1381415	-	3063		V	Acriflavin resistance protein
1381467..1382618	-	1152		V	Putative HlyD family secretion protein
1382794..1384287	-	1494		M	RND efflux system outer membrane lipoprotein NodT family
1384579..1385484	+	906		R	Putative transmembrane protein
1385530..1386333	-	804			hypothetical protein
1386381..1389020	-	2640	*pepN*	E	Aminopeptidase N
1389034..1389912	-	879		R	Transporter drug/metabolite exporter family
1390119..1390841	+	723	*dapB*	E	4-hydroxy-tetrahydrodipicolinate reductase
1390838..1391476	+	639	*rsmG*	J	Ribosomal RNA small subunit methyltransferase G
1392180..1398062	-	5883		S	Putative uncharacterized protein
1398152..1399513	-	1362			Signal transduction histidine kinase LytS
1399589..1400119	-	531			hypothetical protein
1400404..1400559	-	156			hypothetical protein
1400643..1402766	-	2124			Putative uncharacterized protein
1402769..1402930	-	162			hypothetical protein
1402948..1404744	-	1797			Putative uncharacterized protein
1404741..1407917	-	3177	*ntrX*	S	Pentapeptide repeat-containing protein
1407911..1408756	-	846			hypothetical protein
1408991..1409281	-	291			hypothetical protein
1409389..1411617	-	2229	*lepB*	D	Uncharacterized protein
1411756..1411875	-	120			hypothetical protein
1411882..1411995	+	114			hypothetical protein
1412178..1417628	+	5451		T	Ankyrin repeat domain protein
1417816..1421028	+	3213			Uncharacterized protein
1421015..1422688	+	1674			hypothetical protein
1422993..1424408	-	1416	*phrB*	L	Deoxyribodipyrimidine photolyase
1424538..1425200	+	663		C	HAD-superfamily hydrolase subfamily IA variant 1
1425187..1425840	-	654	*ribE*	H	Riboflavin synthase alpha chain
1425843..1427078	-	1236	*kdtA*	M	Three-deoxy-D-manno-octulosonic-acid transferase domain protein
1427229..1428059	+	831	*djlA*	O	DnaJ-like protein DjlA
1428059..1428742	+	684	*rpe*	G	Ribulose-phosphate 3-epimerase
1428744..1429136	+	393			Putative uncharacterized protein
1429175..1431763	+	2589	*lptD*	M	LPS-assembly protein LptD
1431773..1433068	+	1296	*surA*	O	Chaperone SurA
1433098..1434252	-	1155		E	Dipeptide ABC transporter permease component
1434482..1435726	-	1245			hypothetical protein
1439284..1441170	-	1887	*mnmG*	J	tRNA uridine 5-carboxymethylaminomethyl modification enzyme MnmG
1441431..1442186	+	756	*mip*	O	Peptidyl-prolyl cis-trans isomerase
1442366..1442578	+	213	*yjbJ*	S	CsbD family protein
1442630..1443943	-	1314	*tilS*	J	tRNA(Ile)-lysidine synthase
1443998..1444945	-	948	*accA*	I	Acetyl-coenzyme A carboxylase carboxyl transferase subunit alpha
1444948..1445241	-	294			hypothetical protein
1445325..1445909	+	585		L	Uracil-DNA glycosylase
1445906..1446361	-	456		O	Heat shock protein Hsp20
1446628..1446957	+	330		C	Putative ferredoxin 2Fe-2S protein
1446938..1447846	+	909	*htrB*	I	Lipid A biosynthesis (KDO)2-(Lauroyl)-lipid IVA acyltransferase
1447858..1449054	+	1197	*tyrP*	E	Putative tyrosine-specific transport protein
1449076..1450272	+	1197	*tyrP*	E	Tryptophan/tyrosine permease
1450309..1452306	+	1998	*tktA*	G	Transketolase
1452327..1453340	+	1014	*gap*	G	Glyceraldehyde-3-phosphate dehydrogenase type I
1453493..1453723	+	231	*rpoZ*	K	DNA-directed RNA polymerase subunit omega
1453744..1455864	+	2121	*spoT*	T	Guanosine-3' 5'-bis(Diphosphate) 3'-pyrophosphohydrolase
1456022..1457188	+	1167	*speF*	E	Ornithine decarboxylase
1457235..1458287	+	1053		O	Deoxyhypusine synthase-like protein
1458287..1459168	+	882	*speB*	E	Agmatinase
1459216..1459998	-	783	*panC*	H	Pantothenate synthetase
1459982..1460767	-	786	*panB*	H	3-methyl-2-oxobutanoate hydroxymethyltransferase
1461133..1462080	+	948	*waaC*	M	Lipopolysaccharide heptosyltransferase I
1462067..1462588	-	522			hypothetical protein

1462701..1464710	+	2010	*priA*	L	Primosomal protein N	
1464716..1465492	-	777		M	Putative uncharacterized protein	
1465571..1466944	-	1374	*sdhL*	E	L-serine dehydratase	
1467008..1468327	-	1320			hypothetical protein	
1468399..1468827	-	429	*trbN*	M	Soluble lytic murein transglycosylase and related regulatory proteins (some contain LysM/invasin domains)	
1468824..1470218	-	1395	*gadC*	E	Putative amino acid antiporter	
1470226..1470708	-	483	*lspA*	M	Lipoprotein signal peptidase	
1470750..1470875	+	126			hypothetical protein	
1470879..1473725	-	2847	*ileS*	J	Isoleucine--tRNA ligase	
1473824..1474225	-	402	*pilA*	N	Pilus assembly protein PilA	
1474512..1474739	+	228			hypothetical protein	
1475111..1475485	-	375		M	Uncharacterized protein	
1476036..1476218	-	183			hypothetical protein	
1476337..1477341	-	1005		T	Ankyrin repeat domain protein	
1477677..1478231	-	555	*ahpC*	V	Alkylhydroperoxide reductase C	
1478361..1479203	-	843	*ispA*	I	Farnesyl-diphosphate synthase	
1479203..1479445	-	243	*xseB*	L	Exodeoxyribonuclease 7 small subunit	
1479618..1480982	+	1365		R	Peptidase M16 family	
1480972..1482294	+	1323		R	Non-proteolytic protein peptidase family M16	
1482320..1482481	-	162		X	Uncharacterized protein	
1483098..1483457	+	360			hypothetical protein	
1483681..1484991	-	1311	*hemL*	H	Glutamate-1-semialdehyde 2 1-aminomutase	
1485252..1487300	+	2049	*mrdA*	M	Cell elongation specific D D-transpeptidase	
1487436..1487726	+	291	*rpsT*	J	30S ribosomal protein S20	x
1487732..1488586	-	855		R	Uncharacterized protein	
1488603..1489679	-	1077	*ynaI*	M	Putative small-conductance mechanosensitive channel	
1489693..1490328	-	636	*coq7*	H	2-nonaprenyl-3-methyl-6-methoxy-1 4-benzoquinol hydroxylase	
1490416..1491768	+	1353	*htrA*	O	Periplasmic serine protease Do heat shock protein HtrA	
1491928..1493133	-	1206	*coaBC*	H	Phosphopantothenoylcysteine decarboxylase/phosphopantothenate--cysteine ligase	
1493210..1494739	-	1530		M	Putative uncharacterized protein	
1494827..1495552	-	726		P	Integral membrane protein TerC	
1495563..1495877	-	315	*vapD*	S	Putative virulence-associated protein D	
1495885..1496100	-	216			Uncharacterized protein	
1496234..1497613	-	1380		M	Uncharacterized protein	
1497872..1498012	+	141			hypothetical protein	
1498118..1498441	-	324	*ydhD*	O	Glutaredoxin	
1498600..1499184	+	585	*sodB*	P	Superoxide dismutase	
1499223..1500398	+	1176	*argD*	E	Acetylornithine aminotransferase	
1500395..1501570	+	1176	*metB*	E	Cystathionine gamma-lyase	
1501579..1502622	+	1044		J	tRNA methyl transferase-like protein	
1502694..1502999	+	306		O	tRNA 5-methylaminomethyl-2-thiouridine synthase TusA	
1503007..1504140	+	1134	*ydaO*	P	cation efflux family protein	
1504142..1504918	+	777	*xthA*	L	Exodeoxyribonuclease III	
1504928..1505248	+	321	*hspQ*	O	Hemimethylated DNA-binding region	
1505255..1505872	+	618	*sixA*	T	Putative phosphohistidine phosphatase SixA	
1505859..1507184	+	1326		R	methyltransferase domain family	
1507181..1507783	+	603	*engB*	D	Probable GTP-binding protein EngB	
1507795..1507920	+	126			hypothetical protein	
1507964..1508440	-	477			hypothetical protein	
1508490..1509605	-	1116		T	Ankyrin repeat domain protein	
1509852..1511843	+	1992		S	Oligopeptide transporter	
1512056..1513792	+	1737		P	ABC transporter permease protein	
1513846..1515159	+	1314	*tauB*	P	ABC transporter ATP binding protein	
1515174..1516142	+	969	*kdsD*	M	Arabinose 5-phosphate isomerase	
1516182..1516328	+	147			hypothetical protein	
1516318..1516890	+	573	*lptC*	M	Component of the Lpt lipopolysaccharide transport system	
1516899..1517432	+	534	*lptA*	M	Component of the Lpt lipopolysaccharide transport system	
1517429..1518157	+	729	*lptB*	M	Lipopolysaccharide export system ATP-binding protein	
1518267..1518605	+	339	*yggX*	J	Sigma 54 modulation protein/ribosomal protein S30EA	
1518697..1520106	+	1410	*purB*	F	Adenylosuccinate lyase	
1520929..1522626	-	1698			Hypothetical cytosolic protein	
1522668..1523861	-	1194			Uncharacterized protein	
1524161..1526767	-	2607	*clpB*	O	Protein disaggregation chaperone	
1527089..1528048	+	960	*sppA*	O	Protease transmembrane protein	
1528067..1528447	+	381	*pilE*	N	Tfp pilus assembly protein PilE	
1528436..1529383	-	948	*ispH*	I	4-hydroxy-3-methylbut-2-enyl diphosphate reductase	
1530134..1531792	-	1659	*ubiB*	H	Probable protein kinase UbiB	
1531789..1532397	-	609		H	Ubiquinone biosynthesis protein UbiJ contains SCP2 domain	
1532397..1533173	-	777	*ubiE*	H	Ubiquinone/menaquinone biosynthesis C-methyltransferase UbiE	
1533218..1534000	-	783			hypothetical protein	

1534750..1536564	+	1815	*typA*	T	GTP-binding protein	
1536648..1538546	-	1899		R	FOG: Ankyrin repeat	
1538848..1540224	+	1377		J	Amidase Asp-tRNAAsn/Glu-tRNAGln amidotransferase A subunit	
1540340..1541659	-	1320	*ahcY*	H	Adenosylhomocysteinase	
1541656..1542807	-	1152	*metK*	H	S-adenosylmethionine synthase	
1543055..1544347	-	1293		G	Transporter MFS superfamily	
1544344..1545645	-	1302	*phtD*	G	Transporter MFS superfamily	
1545762..1546370	-	609	*tdk*	F	Thymidine kinase	
1546370..1547011	+	642		O	Alkyl hydroperoxide reductase/ Thiol specific antioxidant/ Mal allergen	
1547008..1549329	-	2322			UvrD/REP helicase	
1549510..1549842	-	333	*tusE*	P	Sulfurtransferase	
1550101..1550775	-	675	*yccA*	R	Inner membrane protein yccA	
1550862..1551788	-	927	*murI*	M	Glutamate racemase	
1552637..1553545	-	909	*czcD*	P	Cation diffusion facilitator family transporter	
1553550..1554176	-	627	*lolA*	M	Outer-membrane lipoprotein carrier protein	
1554253..1554963	-	711	*bioD*	H	ATP-dependent dethiobiotin synthetase BioD	
1555888..1557039	-	1152	*bioF*	H	8-amino-7-oxononanoate synthase	
1557024..1558010	-	987	*bioB*	H	Biotin synthase	
1558316..1558792	+	477			hypothetical protein	
1559157..1559327	+	171			hypothetical protein	
1559324..1560229	-	906	*ubiA*	H	4-hydroxybenzoate polyprenyltransferase	
1560381..1561646	+	1266	*bioA*	H	Adenosylmethionine-8-amino-7-oxononanoate aminotransferase	
1561654..1562055	-	402			Ribosomal RNA large subunit methyltransferase A (RRNA(Guanine-N(1)-)-methyltransferase)	
1562174..1562728	-	555		S	Putative uncharacterized protein	
1562939..1563949	-	1011	*rpoS*	K	RNA polymerase sigma factor RpoS	
1564061..1564645	-	585		S	membrane protein DedA family	
1564801..1565556	-	756	*surE*	L	5'-nucleotidase SurE	
1565557..1565868	-	312	*icmD*	U	Component of the Dot/Icm secretion system IcmD (DotP)	
1566568..1567269	-	702		L	Phage SPO1 DNA polymerase-related protein	
1567286..1567531	-	246		R	Hemolysin	
1567706..1568425	+	720	*lipB*	H	Octanoyltransferase	
1568550..1569494	+	945	*lipA*	H	Lipoyl synthase	
1569494..1571512	+	2019	*lssY*	S	PAP2 domain protein	
1571577..1572374	-	798	*apaH*	T	Bis(5'-nucleosyl)-tetraphosphatase symmetrical	
1572383..1573636	-	1254		J	Uncharacterized protein	
1573876..1574064	+	189		S	Uncharacterized protein	
1574103..1575152	+	1050	*rfaF*	M	Glycosyl transferase putative gt9A	
1575153..1576541	-	1389	*mnmE*	J	tRNA modification GTPase MnmE	
1576544..1578163	-	1620	*yidC*	M	Membrane protein insertase YidC	
1578391..1578750	-	360	*rnpA*	J	Ribonuclease P protein component	
1578771..1578905	-	135	*rpmH*	J	50S ribosomal protein L34	x
Ribosomal RNA						
888233..889874	-	1642	*rrs*		16S ribosomal RNA	
884137..887416	-	3280	*rrl*		23S ribosomal RNA	
883767..883886	-	120	*rrf*		5S ribosomal RNA	
1359542..1361183	-	1642	*rrs*		16S ribosomal RNA	
1355446..1358725	-	3280	*rrl*		23S ribosomal RNA	
1355076..1355195	-	120	*rrf*		5S ribosomal RNA	
Transfer RNA						
13432..13504	-	73	*trnT*		Thr tRNA	
86951..87023	+	73	*trnE*		Glu tRNA	
125281..125368	+	88	*trnS*		Ser tRNA	
164099..164182	+	84	*trnL*		Leu tRNA	
278177..278249	-	73	*trnK*		Lys tRNA	
278257..278329	-	73	*trnH*		His tRNA	
278353..278426	-	74	*trnR*		Arg tRNA	
278530..278603	-	74	*trnP*		Pro tRNA	
288962..289043	+	82	*trnL*		Leu tRNA	
295742..295814	+	73	*trnV*		Val tRNA	
295827..295900	+	74	*trnD*		Asp tRNA	
317744..317826	+	83	*trnL*		Leu tRNA	
352085..352173	+	89	*trnS*		Ser tRNA	
352185..352258	+	74	*trnR*		Arg tRNA	
368289..368362	-	74	*trnR*		Arg tRNA	
388001..388074	-	74	*trnM*		Met tRNA	
464588..464661	-	74	*trnP*		Pro tRNA	
492498..492571	-	74	*trnM*		Met tRNA	
507128..507199	+	72	*trnV*		Val tRNA	
514093..514179	-	87	*trnS*		Ser tRNA	
661961..662033	-	73	*trnA*		Ala tRNA	
737047..737118	-	72	*trnQ*		Gln tRNA	

746455..746541	+	87	*trnS*	Ser tRNA
750428..750500	+	73	*trnG*	Gly tRNA
750659..750729	+	71	*trnC*	Cys tRNA
750780..750862	+	83	*trnL*	Leu tRNA
758195..758268	+	74	*trnP*	Pro tRNA
887928..888001	-	74	*trnA*	Ala tRNA
888019..888092	-	74	*trnI*	Ile tRNA
916075..916147	+	73	*trnN*	Asn tRNA
999356..999428	+	73	*trnF*	Phe tRNA
1115806..1115878	-	73	*trnL*	Lys tRNA
1139361..1139434	-	74	*trnM*	Met tRNA
1179012..1179083	-	72	*trnW*	Trp tRNA
1179091..1179162	-	72	*trnT*	Thr tRNA
1179200..1179270	-	71	*trnG*	Gly tRNA
1179403..1179484	-	82	*trnY*	Tyr tRNA
1219273..1219354	-	82	*trnL*	Leu tRNA
1359237..1359310	-	74	*trnA*	Ala tRNA
1359328..1359401	-	74	*trnI*	Ile tRNA
1391625..1391698	+	74	*trnR*	Arg tRNA
1474854..1474926	+	73	*trnT*	Thr tRNA
small RNA				
513983..514079	-	97		signal recognition particle RNA
183994..184397	+	404		tmRNA
589612..590305	+	694	*rnpB*	The RNA component of Ribonuclease P

[1]A, RNA processing and modification; C, Energy production and conversion; D, Cell cycle control, cell division, chromosome partitioning; E, Amino acid transport and metabolism; F, Nucleotide transport and metabolism; G, Carbohydrate transport and metabolism; H, Coenzyme transport and metabolism; I, Lipid transport and metabolism; J, Translation, ribosomal structure and biogenesis; K, Transcription; L, Replication, recombination and repair; M, Cell wall/membrane/envelope biogenesis; N, Cell motility; O, Posttranslational modification, protein turnover, chaperones; P, Inorganic ion transport and metabolism; Q, Secondary metabolites biosynthesis, transport and catabolism; R, General function prediction only; S, Function unknown; T, Signal transduction mechanisms; U, Intracellular trafficking, secretion, and vesicular transport; V, Defense mechanisms; X, prophages, transposons.

[2] x, Genes used for the phylogenetic inference (cf. Fig. 3) and the relative rate tests (cf. Table S3).
