# Supplementary material for: Genomic Insight into Symbiosis-Induced Insect Color Change by a Facultative Bacterial Endosymbiont, “Candidatus Rickettsiella viridis”
Source: mBio. 2018 Jun 12;9(3):e00890-18. doi: 10.1128/mBio.00890-18 (PMC6016236; doi:10.1128/mBio.00890-18)
Supplement: TABLE S2 [file mbo003183938st2.pdf]

TABLE S2 Pseudogenes in the "*Ca.* Rickettsiella viridis" genome.

Location	Strand	Nucleotide length	*Gene*	COG category[1]	Products
10120..10476	+	354	*crcB*	P	Putative fluoride ion transporter CrcB
105770..107342	-	1573	*yhjG*	R	Uncharacterized aromatic compound monooxygenase
125401..125945	-	545		J	2'-5' RNA ligase
222827..224003	+	1177		T	Putative sensory histidine-kinase / response regulator
224432..226103	+	1672			Putative uncharacterized protein
234101..234415	-	315	*ydaD*	X	Transposase
238135..238468	-	334			Uncharacterized protein
239747..240133	-	387		R	partial sequence of ankyrin domain containing protein
265625..266363	+	739	*virB9*	U	type IV secretion system protein VirB9
354235..355075	+	841		X	Transposase IS4 family
385774..386004	-	231		X	Putative transposase
386259..387944	-	1686			Probable activation/secretion signal peptide protein
391770..393230	-	1461	*gcvP*	E	Probable glycine dehydrogenase [decarboxylating] subunit 2
411709..412184	-	476	*ygaD*		CinA C-terminal domain
440531..440656	-	126		X	Putative transposase
484193..485058	+	866	*msrA*	O	Peptide methionine sulfoxide reductase
493094..494947	-	1854		M	Sulfatase
497398..498273	-	876	*yijE*	R	EamA-like transporter family
504716..506694	+	1979	*uvrB*	L	UvrABC system protein B
559616..560485	+	870			Uncharacterized protein
561496..562707	+	1212	*wxcB*	M	LPS O-antigen biosynthesis tyrosine kinase
614660..615923	+	1264	*gshA*	E	Glutamate--cysteine ligase
662141..663188	-	1048		L	Phage integrase
671401..673407	+	2007	*arcB*	T	Putative sensory histidine-kinase / response regulator
707121..707619	+	499	*scpB*	K	Prokaryotic chromosome segregation and condensation protein
747823..749622	+	1800	*uvrC*	L	UvrABC system protein C
750891..751097	-	207	*yoeB*	V	Addiction module toxin
882558..882976	-	419	*ddl*	M	D-alanine--D-alanine ligase
917937..918370	-	434			D-amino-acid:oxygen oxidoreductase
929440..929559	-	120		X	Putative phage integrase
961538..962141	-	604	*pncA*	Q	Nicotinamidase
971342..972424	+	1083		Q	ABC transporter permease
1000318..1001238	+	921	*ydaD*	S	Transposase
1004344..1010146	-	5803		I	Non-ribosomal peptide synthase/Short chain acyl-CoA synthetase/FkbH like protein
1010698..1011069	+	372		T	Putative sensory box histidine kinase/response regulator
1031445..1032728	-	1284			Uncharacterized protein
1053819..1054408	-	590			Uncharacterized protein
1103254..1103992	+	739	*virB9*	U	type IV secretion system protein VirB9
1122573..1123686	-	1114	*ald*	E	Alanine dehydrogenase
1234760..1235578	-	819	*mltD*	M	predicted membrane-bound lytic murein transglycosylase D
1283230..1283550	+	321			Uncharacterized protein
1283907..1284629	-	723	*bamD*	R	competence lipoprotein ComL
1289534..1289953	-	420			Cytochrome C assembly protein
1306113..1307403	+	1291	*rlmD*	J	23S rRNA (uracil(1939)-C(5))-methyltransferase RlmD
1317481..1318020	+	540	*virB1*	U	type IV secretion system protein VirB1
1326572..1327867	-	1296	*pilQ*	U	Type IV pilus biogenesis protein PilQ
1333020..1333758	+	739		R	ABC transporter ATP-binding component
1345844..1347314	-	1471		I	Acetyl-coenzyme A synthetase
1391814..1392102	-	289		X	Transposase
1435826..1439251	-	3426	*mfd*	L	Transcription-repair coupling protein Mfd
1520132..1520902	+	771			Uncharacterized protein
1529413..1529757	-	345		T	Response regulator receiver protein
1529905..1530099	-	195		U	Sec-independent protein translocase protein TatA
1534071..1534591	-	521			Putative uncharacterized protein
1551853..1552622	-	770			Putative uncharacterized protein
1555000..1555868	-	869	*bioC*	H	Malonyl-CoA O-methyltransferase BioC
1566034..1566553	-	520	*fimT*	U	Type 4 pili biogenesis protein (Prepilin-like protein)

[1]Described in Table S1.
