# Supplementary material for: Genomic Insight into Symbiosis-Induced Insect Color Change by a Facultative Bacterial Endosymbiont, “Candidatus Rickettsiella viridis”
Source: mBio. 2018 Jun 12;9(3):e00890-18. doi: 10.1128/mBio.00890-18 (PMC6016236; doi:10.1128/mBio.00890-18)
Supplement: TABLE S3 [file mbo003183938st3.pdf]

TABLE S3 Relative-rate test for comparing the molecular evolutionary rates of 53 concatenated ribosomal protein sequences between "*Ca.* Rickettsiella viridis" and allied endocellular bacteria of the gammaproteobacterial order *Legionellales*.

**Lineage 1**	**Lineage 2**	**Outgroup**	**K1**[1]	**K2**[2]	**K1-K2**	**K1/K2**	***P*-value**[3]
Rickettsiella viridis	*Rickettsiella grylli*	*Coxiella burnetii*	0.098	0.109	-0.011	0.90	0.13
Rickettsiella viridis	*Coxiella burnetii*	*Escherichia coli*	0.257	0.235	0.022	1.09	0.037
Rickettsiella viridis	*Legionella pneumophila*	*Escherichia coli*	0.268	0.229	0.039	1.17	0.0002

[1]Estimated mean distance between lineage 1 and the last common ancestor of lineages 1 and 2.
[2]Estimated mean distance between lineage 2 and the last common ancestor of lineages 1 and 2.
[3]*P*-value was generated using the program RRTree (99).
