# Supplementary material for: Genomic Insight into Symbiosis-Induced Insect Color Change by a Facultative Bacterial Endosymbiont, “Candidatus Rickettsiella viridis”
Source: mBio. 2018 Jun 12;9(3):e00890-18. doi: 10.1128/mBio.00890-18 (PMC6016236; doi:10.1128/mBio.00890-18)
Supplement: TABLE S4 [file mbo003183938st4.pdf]

TABLE S4 Comparison of the gene repertoire between "*Ca.* Rickettsiella viridis" and allied gammaproteobacteria.

COG category	*Rickettsiella viridis*	*Rickettsiella grylli*	*Rickettsiella isopodorum*	*Coxiella burnetii*	*Legionella pneumophila*	*Hamiltonella defensa*	*Buchnera aphidicola*	*Escherichia coli*
Metabolism								
Amino acid transport and metabolism	54	56	62	88	165	73	56	345
Coenzyme transport and metabolism	65	77	77	93	128	81	35	166
Nucleotide transport and metabolism	33	35	35	43	69	41	25	102
Carbohydrate transport and metabolism	38	48	46	65	101	48	30	344
Lipid transport and metabolism	52	46	54	71	142	39	16	118
Energy production and conversion	60	70	69	79	141	58	43	287
Inorganic ion transport and metabolism	31	40	40	42	88	41	16	194
Secondary metabolites transport and metabolism	4	9	11	8	36	40	0	46
Cellular processes								
Cell wall/membrane/envelope biogenesis	123	124	134	147	194	129	30	233
Cell motility	12	13	16	14	70	38	27	115
Cell cycle control, cell division, chromosome partitioning	26	28	24	30	61	43	12	42
Signal transduction mechanisms	47	35	35	56	157	50	9	163
Intracellular trafficking, secretion, and vesicular transport	63	63	53	40	79	104	10	54
Posttranslational modification, protein turnover, chaperones	61	74	73	66	141	65	37	150
Defense mechanisms	17	20	15	31	98	51	7	92
Information storage and processing								
Translation, ribosomal structure and biogenesis	156	164	158	173	221	173	133	240
Transcription	36	35	31	43	93	71	18	283
Replication, recombination and repair	69	73	73	80	115	127	37	139
RNA processing and modification	1	1	1	1	1	1	1	2
Poorly characterized								
General function prediction only	67	57	58	91	193	48	12	191
Function unknown	56	52	48	64	131	71	5	201
Unassigned	291	229	136	452	449	511	3	664
Transposase and phage related	16	25	4	34	49	191	0	72
Total	1378	1374	1253	1811	2922	2094	562	4243
