# Supplementary material for: Genomic Insight into Symbiosis-Induced Insect Color Change by a Facultative Bacterial Endosymbiont, “Candidatus Rickettsiella viridis”
Source: mBio. 2018 Jun 12;9(3):e00890-18. doi: 10.1128/mBio.00890-18 (PMC6016236; doi:10.1128/mBio.00890-18)
Supplement: FIG S1 [file mbo003183938sf1.pdf]

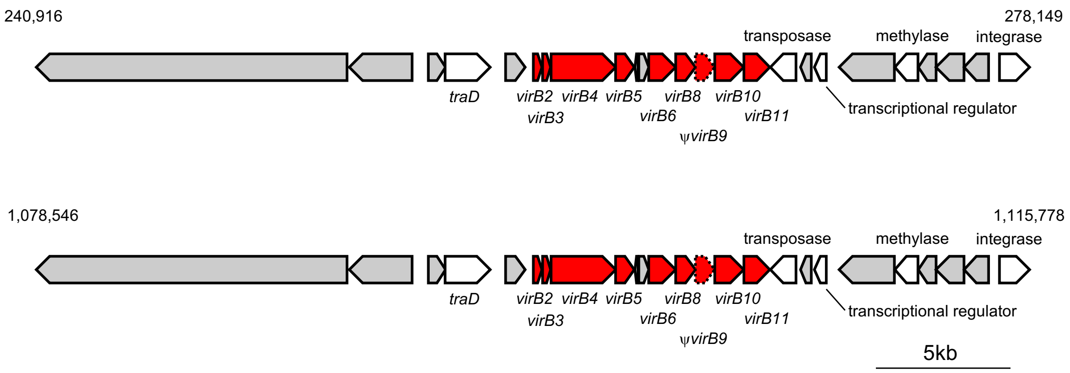

FIG S1 Gene orders of VirB type IV secretory systems encoded in mobile genetic element-like regions in the genome of "*Ca.* Rickettsiella viridis". Pentagons indicate genes, with the coding direction to the tip indicated. Pentagons encircled by a dashed line represent pseudogenes. Red pentagons represent *virB* genes. Gray pentagons represent genes encoding proteins of unknown function. The nucleotide sequences of these two regions are exactly the same.
