# Supplementary material for: Genomic Insight into Symbiosis-Induced Insect Color Change by a Facultative Bacterial Endosymbiont, “Candidatus Rickettsiella viridis”
Source: mBio. 2018 Jun 12;9(3):e00890-18. doi: 10.1128/mBio.00890-18 (PMC6016236; doi:10.1128/mBio.00890-18)
Supplement: TABLE S5 [file mbo003183938st5.pdf]

**TABLE S5** Comparison of secretion systems encoded in the genomes of "*Ca.* Rickettsiella viridis" and allied gammaproteobacteria.

| Secretion system type[1] | *Rickettsiella viridis* | *Rickettsiella grylli* | *Rickettsiella isopodorum* | *Coxiella burnetii* RSA 493 | *Legionella pneumophila* Philadelphia 1 | *Hamiltonella defensa* 5AT | *Buchnera aphidicola* APS | *Escherichia coli* K12 |
|---|---|---|---|---|---|---|---|---|
| Type II secretion system (12) | – | – | – | – | 11 | 10 | – | 12 |
| Type III secretion system (18) | – | – | – | – | – | 11 | – | – |
| Type IV secretion system | | | | | | | | |
| Dot/Icm type (25) | 21 | 20 | 20 | 22 | 25 | – | – | – |
| VirB type (12) | 9[2] | – | – | – | 10 | 9 | – | – |
| Tra type (20) | – | 2 | 2 | – | 8 | 18[2] | – | – |
| Type IV pilus (25) | 11 | 12 | 9 | 12 | 19 | 10 | – | – |
| Flagellar (26) | – | – | – | – | 26 | – | 20 | 26 |

[1]Numbers of parentheses indicate the numbers of protein constituents of the secretion systems represented: Type II secretion system, *E. coli* O78:H11:K80 H10407; Type III secretion system, plasmid pCD1 in *Yersinia pestis*; Dot/Icm, *L. pneumophila* Philadelphia 1; VirB, Ti plasmid in *Agrobacterium tumefaciens*; Tra, plasmid R471 in *Serratia marcesens*; Type IV pilus, *Pseudomonas aeruginosa* PAO1; Flagellar, *E. coli* K12.
[2]Duplicated.
