# Supplementary material for: Genomic Insight into Symbiosis-Induced Insect Color Change by a Facultative Bacterial Endosymbiont, “Candidatus Rickettsiella viridis”
Source: mBio. 2018 Jun 12;9(3):e00890-18. doi: 10.1128/mBio.00890-18 (PMC6016236; doi:10.1128/mBio.00890-18)
Supplement: TABLE S6 [file mbo003183938st6.pdf]

TABLE S6 Genes of "*Ca.* Rickettsiella viridis" encoding putative effector proteins, which are orthologous to effector protein genes identified in *L. pneumophila*.

Position	Coding strand	Gene	Product	Reference[1]
76740–78989	+	*mavN*	Involved in ferrous ion transport	(83, 84)
217452–218255	–	*lpg2628*	Putative transmembrane lipoprotein	(79)
419499–421049	+	*legK1/legK3*	Serine/threonine protein kinase-like	(81)
738885–739754	+	*rsmE* (*lpg2936*)	Ribosomal RNA small subunit methyltransferase E	(79)
1058703–1059512	–	*ravC*	Sporulation protein RMD1-like	(79, 100)
1126677–1127126	–	*lpg2359*	GatB/YqeY domain-containing protein	(79)
1128484–1130475	–	*ravJ*	Hypothetical protein	(79, 100)
1139666–1141066	–	*arp/ankH/sdcA*	Ankyrin repeat-containing protein	(82, 86, 101)
1164066–1166285	–	*sidP* (*lpg0130*)	Phosphoinositide phosphatase	(80)
1409389–1411617	–	*lepB*	Effector protein B	(102)

[1]References 100-102 are below.

100. Huang L, Boyd D, Amyot WM, Hempstead AD, Luo ZQ, O'Connor TJ, Chen C, Machner M, Montminy T, Isberg RR. 2011. The E Block motif is associated with *Legionella pneumophila* translocated substrates. Cell Microbiol 13: 227–245. https://doi.org/10.1111/j.1462-5822.2010.01531.x.

101. Luo ZQ, Isberg RR. 2004. Multiple substrates of the *Legionella pneumophila* Dot/Icm system identified by interbacterial protein transfer. Proc Natl Acad Sci U S A 101:841– 846. https://doi.org/10.1073/pnas.0304916101.

102. Chen J, Reyes M, Clarke M, Shuman HA. 2007. Host cell-dependent secretion and translocation of the LepA and LepB effectors of *Legionella pneumophila*. Cell Microbiol 9:1660 –1671. https://doi.org/10.1111/j.1462-5822.2007.00899.x.
