# Supplementary material for: Genomic Insight into Symbiosis-Induced Insect Color Change by a Facultative Bacterial Endosymbiont, “Candidatus Rickettsiella viridis”
Source: mBio. 2018 Jun 12;9(3):e00890-18. doi: 10.1128/mBio.00890-18 (PMC6016236; doi:10.1128/mBio.00890-18)
Supplement: TABLE S7 [file mbo003183938st7.pdf]

TABLE S7 Genes of "*Ca.* Rickettsiella viridis" encoding proteins with motifs of eukaryotic type.

Position	Coding strand	Motif	Effector
172426–174609	-	Coiled coils	
297385–299613	-	Ser/Thr kinase	
419499–421049	+	Ser/Thr kinase	*legK3*
577881–581027	-	Ankyrin repeats	
584094–585158	+	GMP reductase	
591496–592503	+	Coiled coils	
602039–603064	+	Ser/Thr kinase	
720558–721568	-	Ankyrin repeats	
758862–759233	+	Regulator of chromosome condensation (RCC1) repeats	
810731–811921	-	Tetratricopeptide repeat	
814644–815426	-	Orotidine-5'-phosphate decarboxylase	
840535–841326	-	Ankyrin repeats	
841343–841720	-	Ankyrin repeats	
848659–849666	-	Ankyrin repeats	
930632–931543	-	Coiled coils	
932128–932709	-	Coiled coils	
999284–999451	+	Ankyrin repeats	
1021808–1024756	+	Ankyrin repeats	
1033652–1040023	+	ATPases associated with diverse cellular activities (AAA)	
1073918–1077763	-	Ankyrin repeats	
1123901–1124404	-	Ankyrin repeats	
1139666–1141066	-	Ankyrin repeats	*arp*
1189012–1190718	-	Ankyrin repeats	
1190740–1196613	-	Coiled coils	
1196645–1197760	-	Coiled coils	
1227822–1228718	-	Ras family	
1412178–1417628	+	Ankyrin repeats	
1476337–1477341	-	Ankyrin repeats	
1508490–1509605	-	Ankyrin repeats	
1520929–1522626	-	Ankyrin repeats	
1536648–1538546	-	Ankyrin repeats	
